# An integrated analysis tool for analyzing hybridization intensities and genotypes using new-generation population-optimized human arrays

**DOI:** 10.1186/s12864-016-2478-8

**Published:** 2016-03-31

**Authors:** Mei-Chu Huang, Tzu-Po Chuang, Chien-Hsiun Chen, Jer-Yuarn Wu, Yuan-Tsong Chen, Ling-Hui Li, Hsin-Chou Yang

**Affiliations:** Bioinformatics Program, Taiwan International Graduate Program, Institute of Information Science, Academia Sinica, Taipei, 115 Taiwan; Institute of Statistical Science, Academia Sinica, No 128, Academia Rd, Sec 2, Nankang, Taipei, 115 Taiwan; Institute of Biomedical Informatics, National Yang-Ming University, Taipei, 112 Taiwan; Taiwan International Graduate Program in Molecular Medicine, National Yang-Ming University and Academia Sinica, Taipei, 115 Taiwan; Institute of Biochemistry and Molecular Biology, National Yang-Ming University, Taipei, 112 Taiwan; Institute of Biomedical Sciences, Academia Sinica, Academia Rd, Sec 2, Nankang, Taipei, 115 Taiwan; Institute of Public Health, National Yang Ming University, Taipei, 112 Taiwan; Department of Statistics, National Cheng Kung University, Tainan, 701 Taiwan; Institute of Statistics, National Tsing Hua University, Hsinchu, 300 Taiwan; School of Public Health, National Defense Medical Center, Taipei, 114 Taiwan

**Keywords:** Microarray, Single-nucleotide polymorphism (SNP), Fluorescence intensity, Allele frequency (AF), Allelic imbalance (AI), Loss of heterozygosity (LOH), Long contiguous stretch of homozygosity (LCSH), Copy number variation or alteration (CNV/CNA), Circular binary segmentation (CBS), AF/LOH/LCSH/AI/CNV/CNA Enterprise (ALICE)

## Abstract

**Background:**

Affymetrix Axiom single nucleotide polymorphism (SNP) arrays provide a cost-effective, high-density, and high-throughput genotyping solution for population-optimized analyses. However, no public software is available for the integrated genomic analysis of hybridization intensities and genotypes for this new-generation population-optimized genotyping platform.

**Results:**

A set of statistical methods was developed for an integrated analysis of allele frequency (AF), allelic imbalance (AI), loss of heterozygosity (LOH), long contiguous stretch of homozygosity (LCSH), and copy number variation or alteration (CNV/CNA) on the basis of SNP probe hybridization intensities and genotypes. This study analyzed 3,236 samples that were genotyped using different SNP platforms. The proposed AF adjustment method considerably increased the accuracy of AF estimation. The proposed quick circular binary segmentation algorithm for segmenting copy number reduced the computation time of the original segmentation method by 30–67 %. The proposed CNV/CNA detection, which integrates AI and LOH/LCSH detection, had a promising true positive rate and well-controlled false positive rate in simulation studies. Moreover, our real-time quantitative polymerase chain reaction experiments successfully validated the CNVs/CNAs that were identified in the Axiom data analyses using the proposed methods; some of the validated CNVs/CNAs were not detected in the Affymetrix Array 6.0 data analysis using the Affymetrix Genotyping Console. All the analysis functions are packaged into the ALICE (AF/LOH/LCSH/AI/CNV/CNA Enterprise) software.

**Conclusions:**

ALICE and the used genomic reference databases, which can be downloaded from http://hcyang.stat.sinica.edu.tw/software/ALICE.html, are useful resources for analyzing genomic data from the Axiom and other SNP arrays.

**Electronic supplementary material:**

The online version of this article (doi:10.1186/s12864-016-2478-8) contains supplementary material, which is available to authorized users.

## Background

With the advances in microarray technologies, a whole-genome analysis of genotype and hybridization intensity (HI) data of several million single nucleotide variations and copy number variations (CNVs) has become possible [1, 2]. On the basis of this whole-genome genotype and HI data, the genomic profiles of individual-level allele frequency (AF), allelic imbalance (AI), loss of heterozygosity (LOH), long contiguous stretch of homozygosity (LCSH), copy number alteration (CNAs), and CNVs can be inferred accurately and precisely [3–7]. An integrated analysis of AF, LOH/LCSH, AI, and CNVs/CNAs is useful for characterizing the genomic patterns of individual genomes and identifying typical chromosomal abnormality patterns shared by a group of individuals [8, 9]. For example, cancer genomic studies have analyzed single nucleotide polymorphism (SNP) array data and reported that most of patients with acute lymphoblastic leukemia carry a region of copy-neutral LOH on Chromosome 9; *CDKN2A* (9p21.3), which is located in the LOH region, had a focal hemizygous or homozygous deletion [10–14]. Identifying chromosomal abnormalities enables not only locating disease-susceptibility genes, tumor-suppressor genes, and oncogenes but also deciphering the underlying mechanisms of cancers and other diseases, thus aiding clinical prognosis and prediction and pursuing personalized medicine and targeted cancer treatment [2, 15–22].

An AF indicates the proportion of an allele at a locus and is crucial to genetic and genomic studies. AFs are of two types: “population-level AF,” which reflects the proportion of an allele in a study population, and “individual-level AF,” which reflects the proportion of an allele in a study individual [23]. AI denotes the imbalance status of two alleles resulting from an admixture of heterogeneous cells and can be detected by comparing the relative intensities of the two alleles at a locus [24]. Cell heterogeneity may result from chromosomal abnormalities, such as aneuploidy, gene amplification or deletion, and allelic loss or gain that are frequently observed in cancers, in some of the cells [25–29]. LOH, also called allelic loss, is frequently observed in cancer patients and describes a biological phenomenon whereby the heterozygous status at a genomic locus or region gets altered to a hemizygous or homozygous status. LOH localization, also termed as allelotyping [30], can be performed using a candidate-gene approach [31, 32] or a genome-wide approach [33, 34]. An LCSH is similar to LOH; however, it is observed more frequently in general populations [35, 36]. An LCSH is featured by a continuum of homozygous loci and caused by autozygosity, inbreeding, and evolutionary forces [37, 38].

CNVs indicate DNA variations in the number of copies of genetic loci or gene [39] and have been studied during the past decade [16, 19, 40, 41]. CNAs indicate alterations in copy number (CN) compared with a normal reference, for example, CN loss and gain. CNVs/CNAs, which can range from 1 kilobase (Kb) to several megabases (Mb) or even a whole chromosome, are one of the most abundant structural variations in the human genome. Several large-scale genomic studies on CNVs [16, 17, 19, 42–45] and CNV databases [46–48] have focused on building a blueprint of CNVs in the human genome. CNVs/CNAs have crucial applications in medical and population genomics. In the case of medical genomics, CNVs/CNAs may change gene function, dosage, and expression, thus causing genomic instabilities in cancer patients [49, 50] and increasing disease susceptibility to complex disorders [51–53]. Nevertheless, CNVs/CNAs may also cause interindividual genetic variations and act as genetic markers that silently affect phenotypic changes. In the case of population genomics, CNVs can be used to study genetic backgrounds in global populations [19, 43, 54], examine genetic diversity [55], and infer human evolution on the basis of ancient human genomes [22, 56]. Other applications include but are not limited to anthropological genomics [57, 58] and regenerative medicine [59].

Various biotechnologies have been developed for detecting CNVs/CNAs, with their corresponding strengths and limitations [60, 61]. The approaches comprise target-region approaches (e.g., fluorescent in situ hybridization and spectral karyotyping) and genome-wide approaches [e.g., array-comparative genomic hybridization (a-CGH) and SNP arrays]. The latter approach, which is a powerful untargeted technique for searching CNVs/CNAs without a need of prior information, is becoming increasingly well known. Genome-wide a-CGH provides a higher per-probe signal-to-noise ratio than SNP arrays do. However, an a-CGH experiment requires DNA from both case (patient) and control (matched normal) samples and only provides “locus-specific” hybridization intensities (i.e., total intensity of two alleles at a locus). By contrast, SNP arrays have several advantages. First, a SNP array experiment does not require paired samples. Second, a SNP array can provide both genotype information and allele-specific hybridization intensities. Finally, the marker resolution of SNP arrays is higher than that of a-CGH. These advantages make SNP arrays powerful tools for simultaneously studying CNVs/CNAs and other molecular features such as AF, AI, and LOH/LCSH [15, 56, 62–64].

To account for the genetic heterogeneity in different ethnic populations, new SNP arrays that comprise population-specific SNPs have been recently developed. The Affymetrix Axiom Genome-Wide Population-Optimized Human Array is one of the most well-known population-specific SNP genotyping platforms; Axiom ASI, CEU, CHB, and PanAFR arrays were designed for genetic studies on Asian, Caucasian, Han Chinese, and African populations, respectively. Axiom arrays enable a high-density and high-throughput population-specific genomic analysis at a lower cost and have a promising power because of several merits. First, the Axiom array is the most cost-effective SNP array compared with other whole-genome SNP arrays used today. Therefore, the Axiom array is especially suitable for large-scale whole-genome studies that involve a large number of study individuals. Second, the Axiom array is featured by optimized probe sets from the most recent genomic resources, including the International HapMap project [43, 65, 66], dbSNP database [67], and 1000 Genomes Project [42, 45], to maximize population-specific genomic coverage. Third, 96 or 384 arrays per Axiom plate can be processed together for genotyping experiments. This operational feature can eliminate batch effect, which is caused by different time episodes of an experiment. Finally, the Axiom platform allows for a customized SNP probe set design tailored to different populations such as European [68], East Asian, African American, Latino ethnicity [69], Japanese [70], and other nonhuman species [71–76]. Because of this flexibility, the Axiom array can optimize genome-wide coverage for a targeted population genome and provide a promising whole-genome screening. The Axiom platform has been broadly applied for numerous large-scale genomic studies [77–81]. Axiom arrays have been applied for but are not limited to the mapping of susceptibility loci [77–81], homozygosity mapping [82, 83], anthropological investigation [84–88], pharmacogenomic testing [89], and genome-wide scanning using archived dried blood spot samples [90].

However, unlike other SNP arrays, such as Affymetrix Array 6.0, the Axiom array was originally developed for genotyping but not for CNV/CNA detection. This limitation substantially hindered the applications of the Axiom array in genomic research. The present study will prove that quantitatively analyzing HIs using Axiom arrays is possible and promising. In this paper, we propose new statistical methods for analyzing AF, AI, LOH/LCSH, and CNVs/CNAs on the basis of HI and genotype data from the Affymetrix Axiom Genotyping Solution platforms. We also developed the user-friendly software ALICE (AF/LOH/LCSH/AI/CNV/CNA Enterprise) and genomic reference databases. These achievements will benefit studies that perform integrated analyses of genome-wide AF, AI, LOH/LCSH, and CNVs/CNAs using Affymetrix Axiom Genotyping Solution platforms. ALICE can also analyze data from other Affymetrix gene chips and Illumina bead chips of SNP; however, these additional analysis functions are not emphasized in this paper.

## Results

### Evaluation of proposed coefficient of preferential amplification or hybridization and linear interpolation method adjustment for AF estimation

**Fig. 1:**
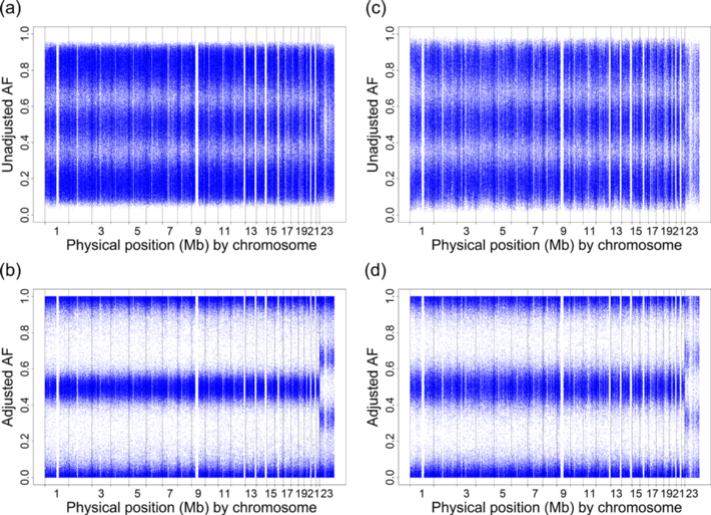
Whole-genome intensity-based AF plots without and with the CPA + LIM adjustment. **a** The plot of the AF estimates of Array 6.0 without the CPA + LIM adjustment; **b** the plot of the AF estimates of Array 6.0 with the CPA + LIM adjustment; **c** the plot of the AF estimates of Axiom without the CPA + LIM adjustment; and **d** the plot of the AF estimates of Axiom with the CPA + LIM adjustment. In each subfigure, each point represents a SNP probe. The vertical axis is the estimated AF, ranging from 0 to 1, and the horizontal axis is the physical position (Mb) on each of the 23 chromosomes

The individual-level AF was estimated by combining the coefficient of preferential amplification or hybridization (CPA) with a linear interpolation method (LIM) (see Individual-level AF estimation with a CPA + LIM adjustment). We illustrate how this CPA + LIM adjustment improved the accuracy of the intensity-based AF estimates. For illustration, in the beginning, we selected a female who was genotyped using both Affymetrix Array 6.0 and Axiom. The results of Array 6.0 were regarded as a benchmark because Array 6.0 designs more SNP probes (approximately 2.86 times) than Axiom does and interrogates CN probes that are not provided by Axiom. This sample had a Chromosome 23 abnormality but no obvious chromosomal aberrations on the 22 autosomes according to the CNVs/CNAs detected on the basis of the Affymetrix Array 6.0 data analysis using the Affymetrix Genotyping Console™ (GTC) software. We first compared the intensity-based AF estimates with and without incorporating the CPA + LIM adjustment across the 22 autosomes. Irrespective of the results of Array 6.0 (Fig. 1a and b) and Axiom (Fig. 1c and d), the adjusted intensity-based AF estimates (Fig. 1b and d) had a clear pattern of three genotypes and were closer to the genotype-based AF estimates than the unadjusted intensity-based AF estimates (Fig. 1a and c). We then examined the sex-chromosome abnormalities. The AF pattern of the allele *A* became much clearer and closer to the ideal AF values of 1 (AAA), 2/3 (AAB), 1/3 (ABB), and 0 (BBB) after applying the proposed CPA + LIM adjustment (Fig. 1b and d).

**Fig. 2:**
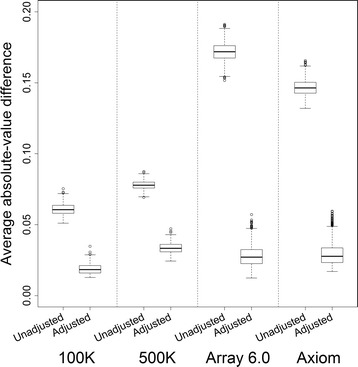
Box-whisker diagrams of the average absolute-value difference between the genotype-based and intensity-based AF estimates. Panels from left to right represent the results of the Affymetrix 100 K (*n* = 367), 500 K (*n* = 448), Array 6.0 (*n* = 1,013), and Axiom (*n* = 1,666) arrays, where *n* is the sample size. In each panel, the box-whisker diagrams on the left- and right-hand sides summarize the results of the AF estimates without the CPA + LIM adjustment (“Unadjusted”) and AF estimates with the CPA + LIM adjustment (“Adjusted”), respectively. Each box-whisker diagram summarizes the distribution of the average absolute-value difference between the genotype- and intensity-based AF estimates of whole-genome SNPs. The extreme values outside the maximum plus a 1.5 interquartile range or the minimum minus a 1.5 interquartile range are indicated by circles

Furthermore, we evaluated the performance of the CPA + LIM adjustment on the basis of a large data set of 2,785 distinct samples that consisted of 367, 448, 1,013, and 1,666 samples genotyped using Affymetrix 100 K, 500 K, Array 6.0, and Axiom, respectively; some of the samples were genotyped using more than one genotyping platform (see Sample materials and genotyping). These samples were qualified as normal samples after carefully examining and were used to construct the ALICE reference databases in this study. Details of the genomic reference databases are described in the ALICE genomic reference databases section. Genotype-based AF estimates, intensity-based AF estimates without a CPA + LIM adjustment, and intensity-based AF estimates with a CPA + LIM adjustment were obtained for each of the 2,785 distinct samples using ALICE. Expectedly, the genomic patterns of the genotype- and intensity-based AF estimates were similar in these normal samples. Box plots indicate that the average absolute-value differences between the genotype- and intensity-based AF estimates substantially decreased after applying the CPA + LIM adjustment, especially for the Array 6.0 and Axiom platforms (Fig. 2). The results demonstrate that the CPA + LIM adjustment improved the accuracy of AF estimation.

In the following subsections, we further evaluate the performance of the ALICE software in the AF, AI, LOH/LCSH, and CNV/CNA analysis. Fifteen additional samples with apparent chromosomal aberrations genotyped using both Array 6.0 and Axiom were characterized. An example of six-panel graphical results obtained using ALICE for an individual is also presented (Additional file 1).

### Whole-genome AF (First panel in the graphical output of ALICE)

**Fig. 3:**
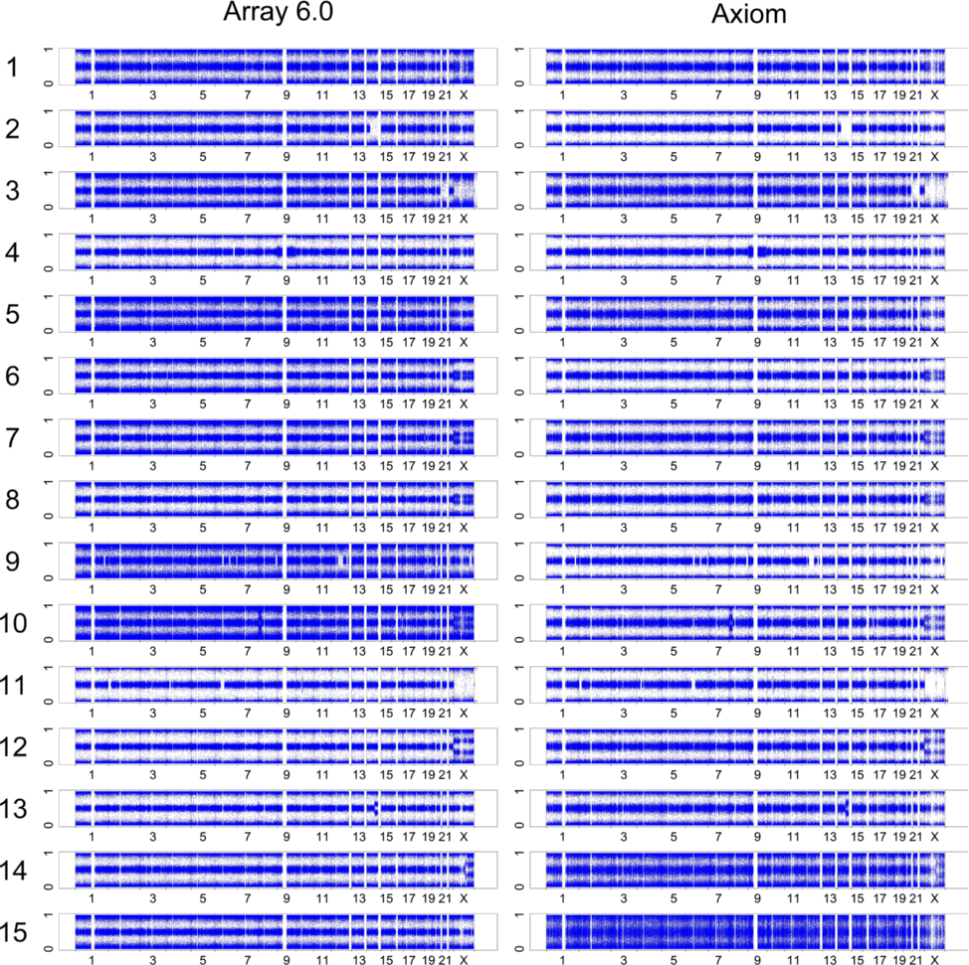
Whole-genome AF plots of 15 individuals genotyped using Array 6.0 and Axiom arrays. The panels of the plot on the left- and right-hand sides are the results of Array 6.0 and Axiom arrays, respectively. In each panel, the whole-genome plots of the intensity-based AF values with the CPA + LIM adjustment of 15 individuals are shown from the top to the bottom, as indicated by the numbers from 1 to 15. In each AF plot, each point represents a SNP probe. The vertical axis is the estimated AF, ranging from 0 to 1, and the horizontal axis is the physical position (Mb) on each of the 23 chromosomes

The whole-genome AF plots of the 15 samples indicate that Array 6.0 and Axiom exhibited similar patterns of AF in the same samples (Fig. 3). In terms of the clarity of patterns of the three genotypes and those of chromosomal aberrations, Axiom was comparable or even more stable than Array 6.0 in analyzing the first 13 samples. These findings imply that Axiom is not only cost effective but also reliable for an intensity-based analysis. Array 6.0 outperformed Axiom in analyzing the 14^th^ and 15^th^ samples; more unexpected noisy points were observed in Axiom than in Array 6.0.

### Detection of AI (Second panel in the graphical output of ALICE)

**Fig. 4:**
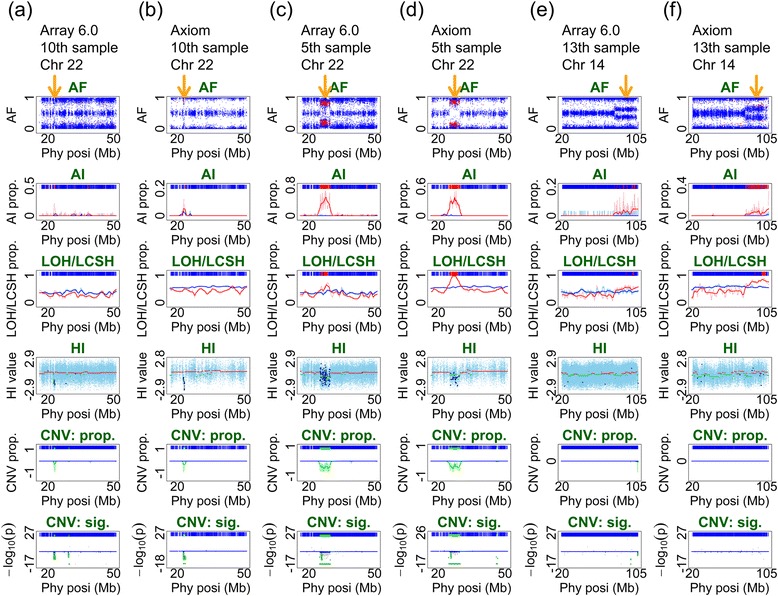
Illustrative examples of the regions of AI identified using both Array 6.0 and Axiom. This figure presents three individuals with relatively small-, medium-, and large-sized regions of AI identified using Array 6.0 and Axiom arrays. The midpoint of an identified region of AI is indicated by an orange arrow on the top of the AF panel of the six subfigures. The figure format of this six-panel plot is provided in Additional file 1. **a** A relatively small-sized region of AI, from 22.54 to 23.63 Mb, on Chromosome 22 is identified in the 10^th^ sample using Array 6.0; **b** A relatively small-sized region of AI, from 22.78 to 23.91 Mb, is identified in the 10^th^ sample using Axiom; **c** A relatively medium-sized region of AI, from 24.58 to 29.98 Mb, on Chromosome 22 is identified in the 5^th^ sample using Array 6.0; **d** A relatively medium-sized region of AI, from 24.03 to 30.34 Mb, is identified in the 5^th^ sample using Axiom; **e** A relatively large-sized region of AI, from 80.18 to 104.81 Mb, on Chromosome 14 is identified in the 13^th^ sample using Array 6.0; **f** A relatively large-sized region of AI, from 80.69 to 107.26 Mb, is identified in the 13^th^ sample using Axiom

Of the 15 samples in Fig. 3, we picked three samples (10^th^, 5^th^, and 13^th^) that carried relatively small, medium, and large regions of AI to demonstrate the performance of Axiom in detecting AI using ALICE. AI was detected using our proposed AI detector (see Single-point index of AI detection and multipoint indices of AI, LOH/LCSH, and CNV/CNA detection section). The 10^th^ sample carried a relatively small region of AI on Chromosome 22, 22.54–23.63 Mb as identified by Array 6.0 (Fig. 4a) and 22.78–23.91 Mb as identified by Axiom (Fig. 4b). The fifth sample carried a relatively medium-sized region of AI on Chromosome 22, 24.58–29.98 Mb as identified by Array 6.0 (Fig. 4c) and 24.03–30.34 Mb as identified by Axiom (Fig. 4d). The 13^th^ sample carried a relatively large region of AI on Chromosome 14, 80.18–104.81 Mb as identified by Array 6.0 (Fig. 4e) and 80.69–107.26 Mb as identified by Axiom (Fig. 4f). The three aforementioned regions of AI were consistently detected not only in the AF plot (first panel) but also in the AI plot (second panel) by both Axiom and Array 6.0 (Fig. 4). The results demonstrate that both Axiom and Array 6.0 can optimally detect regions of AI using ALICE. Notably, the signals of AI in Axiom were stronger than those in Array 6.0 in some cases. For example, the AF plot of Array 6.0 (Fig. 4a) was noisier than that of Axiom (Fig. 4b) for the 10^th^ sample. The noise had considerably weakened the signal of the identified region of AI and increased tiny regions of AI along the chromosome, which was apparently false positive in Array 6.0. Similar noise interference in Array 6.0 was also observed for the 13^th^ sample. The intact region of AI detected by Axiom was consistent with the pattern of AF (Fig. 4f) but divided into numerous small disjoint segments by Array 6.0 (Fig. 4e).

### Detection of LOH/LCSH (Third panel in the graphical output of ALICE)

**Fig. 5:**
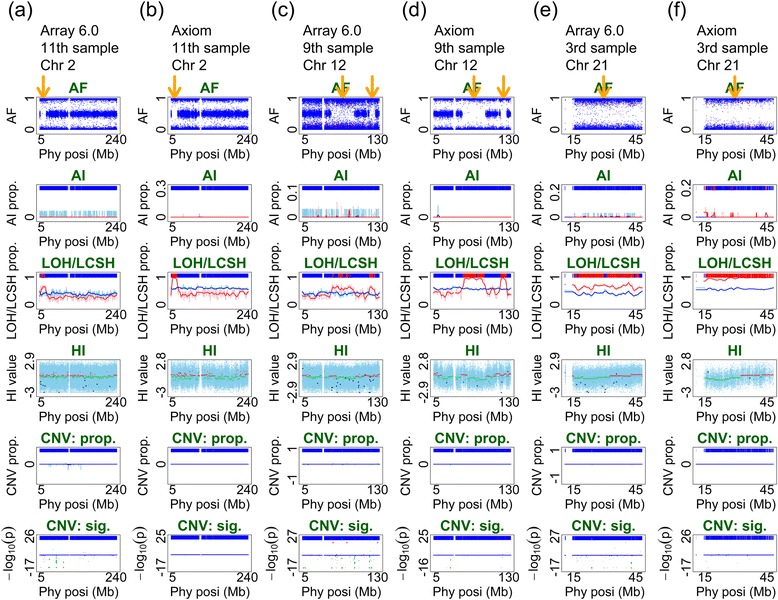
Illustrative examples of the regions of LOH/LCSH identified using both Array 6.0 and Axiom. This figure presents three individuals with regions of LOH/LCSH identified using Array 6.0 and Axiom arrays. The midpoint of an identified region of LOH/LCSH is indicated by an orange arrow on the top of the AF panel of the six subfigures. The figure format of this six-panel plot is provided in Additional file 1. **a** A region of LOH/LCSH, from 2.17 to 20.88 Mb, on Chromosome 2 is identified in the 11^th^ sample using Array 6.0; **b** A region of LOH/LCSH, from 1.88 to 20.88 Mb, is identified in the 11^th^ sample using Axiom; **c** Two regions of LOH/LCSH, from 50.76 to 84.06 Mb and from 115.77 to 126.52 Mb, on Chromosome 12 are identified in the 9^th^ sample using Array 6.0; **d** Two regions of LOH/LCSH, from 48.84 to 89.83 Mb and from 115.53 to 126.71 Mb, are identified in the 9^th^ sample using Axiom; **e** A region of LOH/LCSH, from 14.37 to 47.53 Mb, on Chromosome 21 is identified in the 3^rd^ sample using Array 6.0; **f** A region of LOH/LCSH, from 10.87 Mb to 48.09 Mb, is identified in the 3^rd^ sample using Axiom

Of the 15 samples in Fig. 3, we picked three samples (11^th^, 9^th^, and 3^rd^) that carried LOH/LCSH regions of different lengths to demonstrate the performance of Axiom in detecting LOH/LCSH using ALICE. LOH/LCSH was detected using our proposed LOH/LCSH detector (see Single-point index of LOH/LCSH detection and multipoint indices of AI, LOH/LCSH, and CNV/CNA detection section). The regions that exhibited aberrant patterns in the AF plot (first panel) were also concordantly detected in the LOH/LCSH plot (third panel) by both Axiom and Array 6.0 (Fig. 5). The 11^th^ sample carried a region of LOH/LCSH on Chromosome 2, 2.17–20.88 Mb as identified by Array 6.0 (Fig. 5a) and 1.88–20.88 Mb as identified by Axiom (Fig. 5b). The ninth sample carried two regions of LOH/LCSH on Chromosome 12, 50.76–84.06 Mb and 115.77–126.52 Mb as identified by Array 6.0 (Fig. 5c) and 48.84–89.83 Mb and 115.53–126.71 Mb as identified by Axiom (Fig. 5d). The third sample carried a region of LOH/LCSH ranging from 14.37 to 47.53 Mb, corresponding to the whole q arm of Chromosome 21, as identified by Array 6.0 (Fig. 5e), and from 10.87 to 48.09 Mb as identified by Axiom (Fig. 5f). The results demonstrate that Axiom optimally detected LOH/LCSH. Notably, the pattern of LOH/LCSH in Axiom was more significant than that in Array 6.0. The regions of LOH/LCSH in Array 6.0 (Fig. 5a, c, and e) were interrupted by small proportions of heterozygous SNPs; however, the proportions were higher than those in Axiom (Fig. 5b, d, and f).

### Detection of CNVs/CNAs (Fourth to sixth panels in the graphical output of ALICE)

The three samples for evaluating AI detection (10^th^, 5^th^, and 13^th^) and those for evaluating LOH/LCSH detection (11^th^, 9^th^, and 3^rd^) were also used to evaluate CNV/CNA detection. CNVs/CNAs were detected using our proposed CNV/CNA detector (see Single-point index of CNV/CNA detection and multipoint indices of AI, LOH/LCSH, and CNV/CNA detection section). Array 6.0, which combines SNP and CN probes, identified a region of CN loss from 22.68 to 23.32 Mb on Chromosome 22 in the 10^th^ sample (Fig. 4a), a region of CN loss from 24.84 to 29.88 Mb on Chromosome 22 in the 5^th^ sample (Fig. 4c), and two short regions of CN loss close to the telomere of Chromosome 14 in the 13^th^ sample (Fig. 4e). The results of Axiom also indicated the same regions of CN loss as Array 6.0 did in the 10^th^ and 5^th^ samples; the region in the 10^th^ sample ranged from 22.60 to 23.76 Mb (Fig. 4b) and that in the 5^th^ sample ranged from 24.88 to 29.85 Mb (Fig. 4d). In general, although the density of SNP probes in Array 6.0 is higher than that in Axiom, and CN probes are only included in Array 6.0, the performance of Axiom is comparable to that of Array 6.0 in CNV/CNA detection using ALICE. Nevertheless, a short region with CN loss, 106.6–106.82 Mb, close to the telomere of Chromosome 14q, was detected by Array 6.0 (Fig. 4e), but not by Axiom (Fig. 4f). In this 0.22-Mb region, Array 6.0 designed 95 CN and 5 SNP probes; however, Axiom designed only 9 SNP probes. The CN loss was identified by the CN probes in Array 6.0, but not by SNP probes. Thus, this CN loss was not detected by Axiom. In the 11^th^, 9^th^, and 3^rd^ samples, no CN gain or loss was detected by Array 6.0 (Fig. 5a, c, and e) or Axiom (Fig. 5b, d, and f), implying that the regions of homozygosity resulted from LCSH or copy-neutral LOH rather than deletion-type LOH.

### Consistency in the results of Axiom and Array 6.0 in analyzing a pure tumor tissue sample

**Fig. 6:**
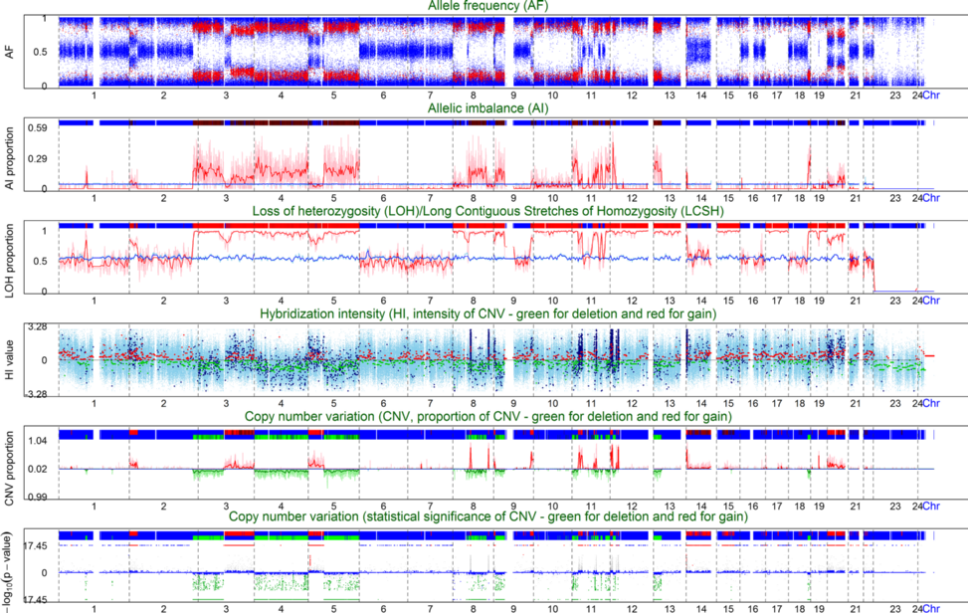
A whole-genome six-panel plot of a cancer cell line sample genotyped using Axiom array. This figure depicts a six-panel plot that shows the graphical results of the AF, AI, LOH/LCSH, and CNV/CNA analyses provided by ALICE. From top to bottom, the six-panel plot consists of the AF plot, AI plot, LOH/LCSH plot, HI and CN segmentation plot, proportion plot of CNV/CNA, and statistical significance plot of CNV/CNA. The details of the illustrations of each plot are provided in Additional file 1

In the previous subsections, we presented several examples to demonstrate that Axiom can be a cost-effective and reliable alternative to Array 6.0 for detecting chromosomal aberrations based on transformed B-cell samples of healthy individuals. Here, we compare the results of Axiom and Array 6.0 based on a whole-genome analysis of a pure tumor tissue sample (see Sample materials and genotyping section). We genotyped the pure tumor tissue sample and compared the results of Axiom (Fig. 6) and Array 6.0 (Additional file 2). The results of Axiom and Array 6.0 were highly consistent (Fig. 6; Additional file 2). Moreover, Axiom provided clearer patterns of AF and finer locations of chromosomal aberrations than Array 6.0 did. For a further numerical comparison, we defined the consistency rate of CNV/CNA detection as follows: the consistency rate *C* was defined as the proportion of lengths of the regions that they were identified by Axiom and had an overlap ratio of ≥ 50 % with the regions identified by Array 6.0. Overall, the regions identified by Axiom and Array 6.0 were highly consistent (*C* = 93.8 %). The consistency rates were as high as 97.1 % if we focused on the regions of ≥ 0.5 Mb and further increased to 98.5 % if we focused on the regions of ≥ 1 Mb. The overlap ratio tended to increase with the length of the overlapped region (Additional file 3).

### AF, AI, LOH/LCSH, and CNV/CNA analysis of admixed samples of tumor cells and corresponding normal cells

An experiment involving admixed samples was designed to investigate how Axiom performs in detecting chromosomal aberrations. Admixed samples were prepared by mixing *p*% of the DNA from the cancer cell line and (100 - *p*)% of the DNA from the corresponding blood cell line, and the admixture proportion *p*% ranged from 0 to 100 with an increment of 10 (see Sample Materials and Genotyping). Each sample was genotyped using Axiom and analyzed using ALICE. Dynamic patterns of whole-genome HI (Additional file 4) and AF (Additional file 5) across 11 admixture proportions were observed. The results revealed that in many cases, AI or LOH/LCSH acted as a precursor of CNV/CNA. For example, the sample containing 100 % pure tumor tissue underwent a large CN loss in whole Chromosome 3p and a large CN gain in whole Chromosome 3q. AI and LOH/LCSH were observed when *p*% ≥ 20 %; however, CNVs/CNAs became detectable when *p*% ≥ 30 %.

We also deciphered the interrelationship between the successful detection of chromosomal aberrations and three influential factors: (1) admixture proportion, (2) mean difference in HI values between the study sample and normal reference, and (3) the length of the region of CNVs/CNAs. We first identified regions of CN loss and gain combined with AI or LOH/LCSH on the basis of the pure tumor tissue sample (i.e., *p*% = 100 %). In total, 553 regions of CNVs/CNAs were identified, which comprised 176 and 377 regions of CN gain and loss, respectively. This result was considered a benchmark for the other analyses at different admixture proportions because the cancer cell line sample was not contaminated by normal cells. We defined a successful detection rate *S*(*p*) of chromosomal aberrations at an admixture proportion of *p*% as a ratio of the lengths of regions that were simultaneously identified at an admixture proportion of *p*% and in the pure tumor tissue sample.

**Fig. 7:**
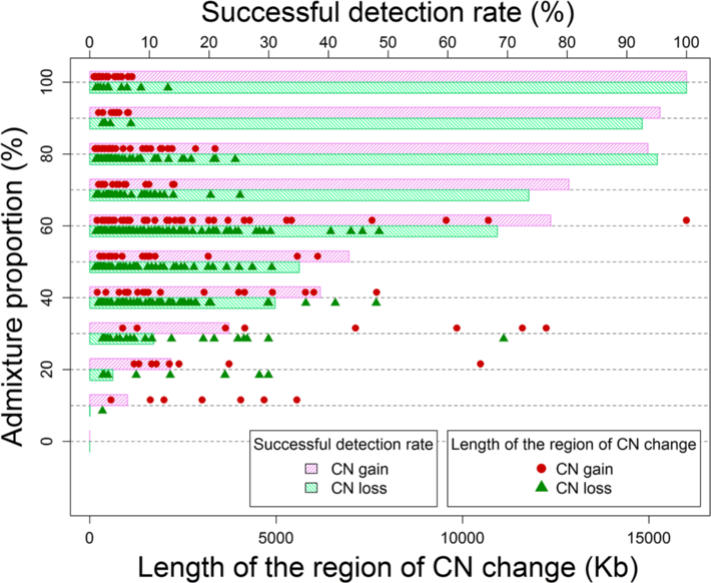
Successful detection rates of CN change in the admixed samples genotyped using Axiom array. The vertical axis indicates the admixture proportion (%), and the horizontal axis is the successful detection rate (%) (top) and the length of the region of CN loss and gain (Kb) (bottom) in the pure cancer cell line sample. Under an admixture proportion, the proportion of successfully detected regions among all 176 (377) regions of CN gain (loss) is presented by a pink right (green left) hatched bar, where the value on the bar represents the coordinate of the top horizontal axis. For each of the 176 (377) regions of CN gain (loss), the minimum admixture proportions that were successfully detected are presented by pink (green) points, where the value of the minimum admixture proportion is represented by the coordinate of the vertical axis, and length (Kb) of the region by the coordinate of the bottom horizontal axis

First, *S*(*p*) increased with increasing *p*%. For CN gain, *S*(*p*) increased from 6.34 to 95.55 % as *p*% was increased from 10 to 90 % (pink right hatched bars in Fig. 7). The increasing trend of *S*(*p*) had a positive slope coefficient of 1.25 × 10^−2^ [standard error (se) = 3.49 × 10^−3^], and the *p* value was 3.53 **×** 10^−4^ for the linear regression of *S*(*p*) on *p*%. For CN loss, *S*(*p*) increased from 0.06 to 92.58 % as *p*% was increased from 10 to 90 % (green left hatched bars in Fig. 7). The increasing trend of *S*(*p*) had a positive slope coefficient of 1.26 **×** 10^−2^ (se **=** 1.31 **×** 10^−3^), and the *p* value was 2.78 **×** 10^−21^ for the linear regression.

Second, *S*(*p*) was positively and negatively correlated with the differences in HI for CN gain and loss, respectively; here, the difference is that the HI of a study sample subtracts the average HI of reference samples in ALICE at each SNP probe. For CN gain, *S*(*p*) increased with an increasing difference in HI; the increasing trend of *S*(*p*) had a positive slope coefficient of 3.15 (se = 3.39 × 10^−1^), and the *p* value was 3.43 × 10^−19^ for the linear regression of *S*(*p*) on the difference in HI. For CN loss, *S*(*p*) increased as the difference in HI became more negative, with a positive slope coefficient of 2.07 (se = 2.30 × 10^−1^), and the *p* value was 6.18 × 10^−19^ for the linear regression.

Finally, *S*(*p*) was also positively correlated with the length of the region of CNVs/CNAs. For CN gain, *S*(*p*) increased with an increase in the length of the region of CNVs/CNAs; the increasing trend of *S*(*p*) had a positive slope coefficient of 0.29 (se = 2.02 × 10^−2^), and the *p* value was 4.18 × 10^−40^. For CN loss as well, *S*(*p*) increased with an increase in the length of the region of CNVs/CNAs; the increasing trend of *S*(*p*) had a positive slope coefficient of 0.19 (se = 1.38 × 10^−2^), and the *p* value was 6.05 × 10^−40^.

We also examined the minimum admixture proportion that enables a region of chromosomal aberration to be detected (*p*_*min*_%). We investigated how *p*_*min*_ relates to the difference in HI values and length of the region of CNVs/CNAs. The results first indicated that *p*_*min*_ was negatively correlated with the difference in HI values. For a CN gain, *p*_*min*_ decreased with an increase in the difference in HI values (Spearman correlation coefficient was −0.53, with a *p* value of 5.44 × 10^−14^). For a CN loss, *p*_*min*_ decreased as the difference in HI values became more negative (Spearman correlation coefficient was −0.16, with a *p* value of 1.75 × 10^−3^). Second, *p*_*min*_ was negatively correlated with the length of the region of CNVs/CNAs when the admixture proportion was higher than 50 % (Fig. 7). For a CN gain, *p*_*min*_ decreased with an increase in the length of the region of CNVs/CNAs (Spearman correlation coefficient was −0.54, with a *p* value of 9.03 × 10^−15^) (solid circles in Fig. 7). For a CN loss, *p*_*min*_ decreased with an increase in the length of the region of CNVs/CNAs (Spearman correlation coefficient was −0.18, with a *p* value of 3.89 × 10^−4^) (solid triangles in Fig. 7).

### Paired-sample analysis

In the previous subsections, we focused on an unpaired-sample analysis. ALICE also provides a paired-sample analysis function (see Methods). In this subsection, we evaluated the performance of Axiom in conducting the paired-sample analysis of ALICE. We reanalyzed the admixture samples, with the corresponding blood cell line sample as a matched control. The six-panel plot of the cancer cell line sample is provided in Additional file 6. The analysis of the pure tumor tissue sample revealed 403 regions of CNVs/CNAs in total, which comprised 127 and 276 regions of CN gain and loss, respectively. Dynamic patterns of whole-genome HI (Additional file 7) and AF (Additional file 8) among different admixture proportions are presented. In general, the results of the paired-sample analysis (Additional files 6, 7 and 8) revealed patterns similar to but not identical from those revealed by the unpaired-sample analysis (Fig. 6; Additional files 4 and 5), for example, the fifth and sixth quantitative polymerase chain reaction (qPCR)-validated regions (see Real-time qPCR validation section). Similar to the unpaired-sample analysis, we deciphered the interrelationship between *S*(*p*) and the aforementioned three influential factors, and also examined how *p*_*min*_ relates to the difference in HI values and length of the region of CNVs/CNAs. The results showed the patterns in the paired-sample and unpaired-sample analysis were similar (Additional file 9).

### Real-time qPCR validation

**Fig. 8:**
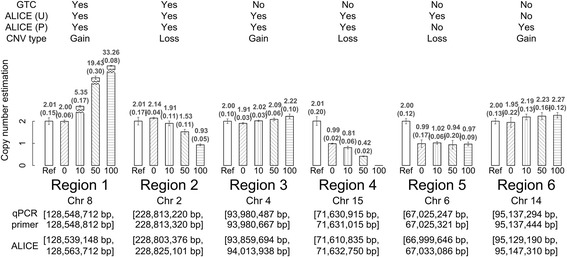
Validation of CNV/CNA using real-time qPCR. Bar charts of CN estimates in six genomic regions are displayed. The bar chart for each genomic region contains five bars, which show the mean CN estimate in the reference sample (“Ref,” white open bar), the pure corresponding blood sample (“0,” right hatched bar), admixed sample of 10 % (“10,” vertical hatched bar), admixed sample of 50 % (“50,” left hatched bar), and the pure cancer cell line sample (“100,” horizontal hatched bar). An error bar represents 1 se of the mean CN estimate. The value of the mean (se) CN estimate is indicated on the top of each bar. The vertical axis represents the value of CN estimate. The index and chromosome of each genomic region are labeled at the bottom of the bar chart, followed by the physical positions of the qPCR primer (labeled as “qPCR primer”) and CNV/CNA region detected using ALICE (labeled as “ALICE”). The starting and ending positions are the first and second elements enclosed in a square bracket. For example, the starting and ending positions of the qPCR primer of the first region are 128,548,712 and 128,548,812 bp. The detection results obtained using GTC (“GTC”), unpaired analysis in ALICE [“ALICE (U)”], and paired analysis in ALICE [“ALICE (P)”] and the type of CNVs/CNAs are labeled at the top of the bar chart

We selected six genomic regions of CNVs/CNAs detected by ALICE for validation using real-time qPCR on the basis of 11 admixed samples and 6 healthy controls (see Real-time qPCR in the Materials and methods section). Physical positions of the six genomic regions and the results of qPCR are presented in Fig. 8. Regions 1 and 2 were consistently detected by the unpaired- and paired-sample analyses of ALICE, as well as by the Affymetrix GTC software (http://www.affymetrix.com/estore/browse/level_seven_software_products_only.jsp?productId=131535#1_1). The GTC can analyze Array 6.0 data; however, it cannot provide a CNV/CNA analysis for Axiom data. Region 1, ranging from 128,539,148 to 128,563,712 (24.564 Kb) on Chromosome 8, contained 16 SNPs. Both the unpaired- and paired-sample analyses identified this region as a CN gain for all admixture proportions of *p*% ≥ 10 %. In the qPCR assay, the average CN estimate of the six reference samples was 2.01 (se = 0.15). The CN estimates of the admixed samples at *p*% = 0 %, 10 %, 50 %, and 100 % were 2.00 (se = 0.06), 5.35 (se = 0.17), 19.43 (se = 0.30), and 33.26 (se = 0.08), respectively. The CN estimates gradually increased with an increase in the admixture proportion. Region 2, ranging from 228,803,376 to 228,825,101 (21.725 Kb) on Chromosome 2, carried 14 SNPs. Both the unpaired- and paired-sample analyses identified this region as a CN loss for all *p*% ≥ 50 %. In the qPCR assay, the average CN estimate of the six reference samples was 2.01 (se = 0.17). The CN estimates of the admixed samples at *p*% = 0 %, 10 %, 50 %, and 100 % were 2.14 (se = 0.04), 1.91 (se = 0.11), 1.53 (se = 0.11), and 0.93 (se = 0.05), respectively. The CN estimates decreased with a gradual increase in the admixture proportion.

Regions 3 and 4 were only detected by the unpaired- and paired-sample analyses of ALICE, but not by GTC. Region 3, ranging from 93,859,694 to 94,013,938 (154.244 Kb) on Chromosome 4, carried 17 SNPs. Both the unpaired- and paired-sample analyses identified this region as a CN gain at *p*% ≥ 90 %. In the qPCR assay, the average CN estimate of the six reference samples was 2 (se = 0.10). The CN estimates of the admixed samples at *p*% = 0 %, 10 %, 50 %, and 100 % were 1.91 (se = 0.03), 2.02 (se = 0.03), 2.09 (se = 0.06), and 2.22 (se = 0.10), respectively. The CN estimates increased with a gradual increase in the admixture proportion. Region 4, ranging from 71,610,835 to 71,632,750 (21.915 Kb) on Chromosome 15, carried 11 SNPs. The unpaired-sample analysis identified this region as a CN loss at *p*% ≥ 20 %. The paired-sample analysis also identified this region as a CN loss at *p*% ≥ 30 %. In the qPCR assay, the average CN estimate of the six reference samples was 2.01 (se = 0.20). The CN estimates of the admixed samples at *p*% = 0 %, 10 %, 50 %, and 100 % were 0.99 (se = 0.02), 0.81 (se = 0.06), 0.42 (se = 0.02), and approximately 0 (se = 0) (i.e., a homozygous deletion), respectively. The CN estimates gradually decreased with an increase in the admixture proportions.

Regions 5 and 6 were detected only by the unpaired- or paired-sample analysis of ALICE, but not by GTC. Region 5, ranging from 66,999,646 to 67,033,086 (33.440 Kb) on Chromosome 6, carried 14 SNPs. This region was detected only by the unpaired-sample analysis but not by the paired-sample analysis or GTC. The unpaired-sample analysis identified this region as a CN loss at all admixture proportions (i.e., *p*% ≥ 0 %). In the qPCR assay, the average CN estimate of the six reference samples was 2 (se = 0.12). The CN estimates of the admixed samples at *p*% = 0 %, 10 %, 50 %, and 100 % were 0.99 (se = 0.17), 1.02 (se = 0.06), 0.94 (se = 0.20), and 0.97 (se = 0.09), respectively. The paired-sample analysis did not identify this region as CN loss, because this one-copy loss had occurred since *p*% = 0 %. Thus, the admixed samples themselves did not have any CNVs/CNAs; however, they exhibited a CN loss relative to the six reference samples. The result suggests that this CNV/CNA is caused by germline mutations.

The final region (Regions 6), ranging from 95,129,190 to 95,147,310 (18.120 Kb) on Chromosome 14, carried 11 SNPs. This region was detected only by the paired-sample analysis but not by the unpaired-sample analysis or GTC. The paired-sample analysis identified this region as a CN gain when *p*% ≥ 70 %. In the qPCR assay, the average CN estimate of the six reference samples was 2 (se = 0.13). The CN estimates of the admixed samples at *p*% = 0 %, 10 %, 50 %, and 100 % were 1.95 (se = 0.22), 2.19 (se = 0.13), 2.23 (se = 0.16), and 2.27 (se = 0.12), respectively. The CN estimates gradually increased with an increase in the admixture proportion. This cancer patient sample had a relatively lower CN intensity at *p*% = 0 % than the mean CN intensity of the six reference samples. This explains why this CN gain was detected by the paired-sample analysis but not by the unpaired-sample analysis. The CN intensities of this cancer patient sample at *p*% = 0 % did not differ significantly from those of the six reference samples, implying that the CNV/CNA is because of somatic and not germline mutations.

### CN segmentation

We analyzed the 11^th^ sample in Fig. 3 as an example to illustrate the ideas of the original CBS algorithm [91, 92] and quick CBS algorithm in ALICE (see CN segmentation in the Materials and methods section). This sample carried a region of LCSH ranging from 1.88 to 20.88 Mb on Chromosome 2 (Additional file 10A and B). The original CBS algorithm required 88 s for the segmentation, which resulted in numerous short segments of the LCSH and non-LCSH regions (the third panel in Additional file 10A). By contrast, the quick CBS algorithm required only 9 s for the segmentation. The LCSH region was separated into several segments, and the non-LCSH regions downstream of the position of 20.88 Mb were grouped into the same segment (the fourth panel in Additional file 10A). A detailed analysis of the LCSH region reveals that the segments obtained by the original CBS algorithm (the third panel in Additional file 10B) and quick CBS algorithm (the fourth panel in Additional file 10B) have a reasonably consistent pattern.

We further compared the performance of the original and quick CBS algorithms in terms of computational time, the number of segments, and the length of segments on the basis of 11 admixed samples from a cancer patient mentioned in the previous subsections and 15 noncancerous samples with apparent chromosomal aberrations shown in Fig. 3. The analysis of the 11 admixed samples revealed that the average computational time per sample was 1.46 and 0.47 min for the original and quick CBS algorithms, respectively. The average number of segments was 1,351.73 and 350.53 for the original and quick CBS algorithms, respectively, and the average length of segments was 2.19 and 21.9 Mb, respectively. The analysis of the data set of the 15 noncancerous samples revealed that the average computational time per sample was 1.50 and 1.04 min for the original and quick CBS algorithms, respectively. The average number of segments was 1,580.73 and 911.09 for the original and quick CBS algorithms, respectively, and the average length of segments was 1.82 and 3.56 Mb, respectively.

### Simulation procedures

**Fig. 9:**
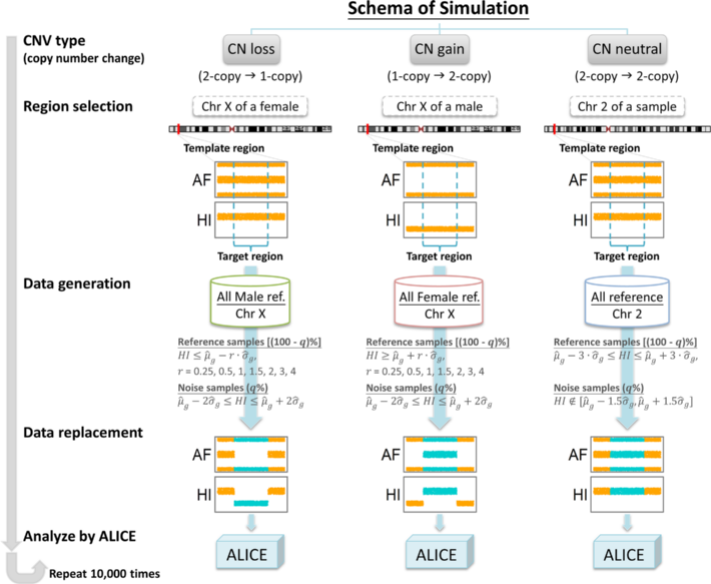
A schema of the simulation study

We conducted a simulation study to evaluate the performance of Axiom arrays in detecting CNVs/CNAs using the ALICE software. To mimic real data structures, genotypes and HI values were generated on the basis of real genotypes and HI values of 1,666 normal samples genotyped using Axiom (The genome assembly of GRCh37/hg19 was employed). These samples were used to construct the Axiom reference databases in the ALICE software (see ALICE genomic reference databases section). Our simulation study considered several simulation parameters for generating data, including the type of CNVs/CNAs, effect size, and admixture proportion. In addition, we considered several settings for CNV/CNA detection, including window sizes (i.e., the number of SNPs in a sliding window) and the numbers of consecutive significant SNPs in a sliding window. In total, 10,000 simulations were performed to evaluate the false positive rate (FPR) (i.e., type 1 error) and true positive rate (TPR) (i.e., power) of a single-point and multipoint CNV/CNA analysis. The schema of this simulation study is depicted in Fig. 9.

First, we considered three scenarios of CNVs/CNAs that ALICE aimed to detect: CN loss, CN gain, and CN neutral. The scenarios of CN loss and gain were designed to evaluate the TPRs of our CNV/CNA detection, and the scenario of CN neutral was designed to evaluate the FPR. We designed a “template region” of 2,001 SNPs for each of the three scenarios of CNV/CNA as follows: (1) for detecting CN loss, we chose a non-pseudo-autosomal region of the p-arm of chromosome X of a female (two copies); (2) for detecting CN gain, we chose a non-pseudo-autosomal region of chromosome X of a male (one copy); and (3) for detecting CN neutral, we chose a region of Chromosome 2 of a male or female (two copies). A shorter “target region” containing *N*_*T*_ = 11, 51, 101, and 501 CN-loss, CN-gain, and CN-neutral SNPs was arranged in the middle of the template region of 2,001 SNPs.

Second, genotypes and HI values were generated under different effect sizes, *r*. Let ${\hat{\mu}}_{i,g}$ and ${\hat{\sigma}}_{i,g}$ denote the genotype-specific sample mean and standard deviation of HI values of the *i*^th^ SNP with genotype *g* in the genomic reference databases of ALICE. The procedures for data generation for the three scenarios of CNVs/CNAs are described as follows: (1) In the scenario of CN loss, genotypes and HI values of a SNP from female samples (two copies) were replaced by genotypes and HI values of the same SNP from male samples (one copy) in the target region on chromosome X. Only variations with one copy and HI values satisfying $\le {\hat{\mu}}_{i,g}-r\cdot {\hat{\sigma}}_{i,g}$ were collected; (2) In the scenario of CN gain, genotypes and HI values of a SNP from male samples (one copy) were replaced by genotypes and HI values of the same SNP from female samples (two copies) in the target region on chromosome X. Only variations with two copies and HI values satisfying $\ge {\hat{\mu}}_{i,g}+r\cdot {\hat{\sigma}}_{i,g}$ were collected. In these two scenarios, we considered *r* = .25, 0.5, 1, 1.5, 2, 3, and 4. The larger the value of *r*, the larger was the effect size; (3) In the scenario of CN neutral, the effect size should be zero (i.e., *r* = 0). Genotypes and HI values of a SNP from male or female samples (two copies) were randomly replaced by genotypes and HI values of the same SNP from other samples (two copies) in the target region. To exclude outliers, only SNPs with two copies and HI values ranging from ${\hat{\mu}}_{i,g}-3\cdot {\hat{\sigma}}_{i,g}$ to ${\hat{\mu}}_{i,g}+3\cdot {\hat{\sigma}}_{i,g}$ were collected.

Finally, in a practical situation, cell admixture (e.g., normal cell contamination - tumor cells are contaminated with adjacent normal cells) or noise interference because of environmental or other uncontrollable factors may occur in sample preparation. Let *q*% denote the proportion of an admixture with normal cell contamination or noise interference in this simulation. The larger the proportion of noise interference, the higher will be the data variation. For example, we generated the HI values for an admixture of a one-copy loss and copy neutral by mixing data from the following two sources: (1) (100 - *q*)% of HI values were sampled from the SNPs on chromosome X of a male, and their HI values were $\le {\hat{\mu}}_{i,g}-r\cdot {\hat{\sigma}}_{i,g}$ and (2) *q*% of HI values were sampled from the SNPs on chromosome X of a male, and their HI values ranged from ${\hat{\mu}}_{i,g}-2\cdot {\hat{\sigma}}_{i,g}$ to ${\hat{\mu}}_{i,g}+2\cdot {\hat{\sigma}}_{i,g}$. We considered the simulation experiments without noise interference (*q*% = 0 %) and with noise interference (*q*% = 25 %). Original genotypes and HI data in the target region of *N*_*T*_ SNPs were replaced by the newly generated genotypes and HI data.

ALICE provides single-point and multipoint CNV/CNA analysis. For a multipoint CNV/CNA analysis, a sliding-window approach was employed to scan CNVs/CNAs in the human genome chromosome by chromosome. We considered four window sizes in the multipoint CNV/CNA analysis: *w* = 11, 51, 101, and 501. CNVs/CNAs that involve consecutive significant SNPs may be more reliable than CNVs/CNAs that have only a single significant SNP. We considered CNVs/CNAs that contain at least the number of consecutive significant SNPs (*n*_*c*_) = 1, 2, 3, 4, and 5 in the multipoint CNV/CNA analysis. The case *n*_*c*_ = 1 means that the CNV/CNA signal is not restricted to several consecutive SNPs.

On the basis of the aforementioned simulation parameters and settings of the CN detection method, we calculated the SNP-level and region-level FPR and TPR in a single-point and multipoint CNV/CNA analysis, respectively. Here, a SNP-level FPR was calculated as a proportion of the event that a CN-neutral SNP was wrongly identified as a CNV/CNA in 10,000 simulations. A SNP-level TPR was calculated as a proportion of the event that a true CN-loss (CN-gain) SNP was correctly identified as a loss (gain) in 10,000 simulations. The region-level FPR and TPR were further calculated by averaging the SNP-level FPRs and TPRs for SNPs in a target region, respectively.

### Simulation results

#### FPR of single-point CN detection

**Fig. 10:**
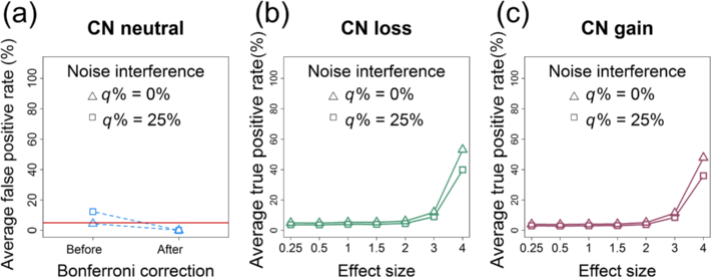
Average FPRs and TPRs of single-point CNV/CNA analyses. **a** The average FPR (%) before and after a Bonferroni correction under the CN-neutral scenario. The horizontal reference line represents an FPR of 0.05. **b** The average TPRs (%) for different effect sizes under the CN-loss scenario. The horizontal axis indicates the effect size (*r* = 0.25 to 4). **c** The average TPRs (%) for different effect sizes under the CN-gain scenario. The horizontal axis indicates the effect size (*r* = 0.25 to 4). Empty triangles and squares indicate the results for the data without noise interference (*q*% = 0 %) and with noise interference (*q*% = 25 %), respectively

The results of FPRs in the single-point CNV/CNA analysis are summarized in Fig. 10a. Noise interference inflated the FPRs. The FPRs exceeded the significance level of 0.05 before a Bonferroni correction; however, they became stable and were under control after a Bonferroni correction. Therefore, our subsequent investigations only focused on the results that had Bonferroni corrections.

#### TPR of single-point CN detection

The results of TPRs in the single-point CNV/CNA analysis for CN loss and gain are summarized in Fig. 10b and c, respectively. First, the TPR increased with an increase in the effect size. For example, for CN loss, when the effect size increased from *r* = 0.25 to 4, the average TPRs increased from 6.24 to 50.37 % under noise interference of *q*% = 0 % and from 4.73 to 37.82 % under *q*% = 25 %. Similarly, for CN gain, when the effect size increased from *r* = 0.25 to 4, the average TPRs increased from 4.56 to 51.65 % under *q*% = 0 % and from 3.42 to 38.82 % under *q*% = 25 %. Finally, the TPRs decreased with an increase in the proportion of noise interference (Fig. 10b and c). For example, for CN loss and effect size *r* = 4, when *q*% increased from 0 to 25 %, the average TPRs decreased from 50.37 to 37.82 %. Similarly, for CN gain, when *q*% increased from 0 to 25 %, the average TPRs decreased from 51.65 to 38.82 %.

#### FPR of multipoint CN detection

**Fig. 11:**
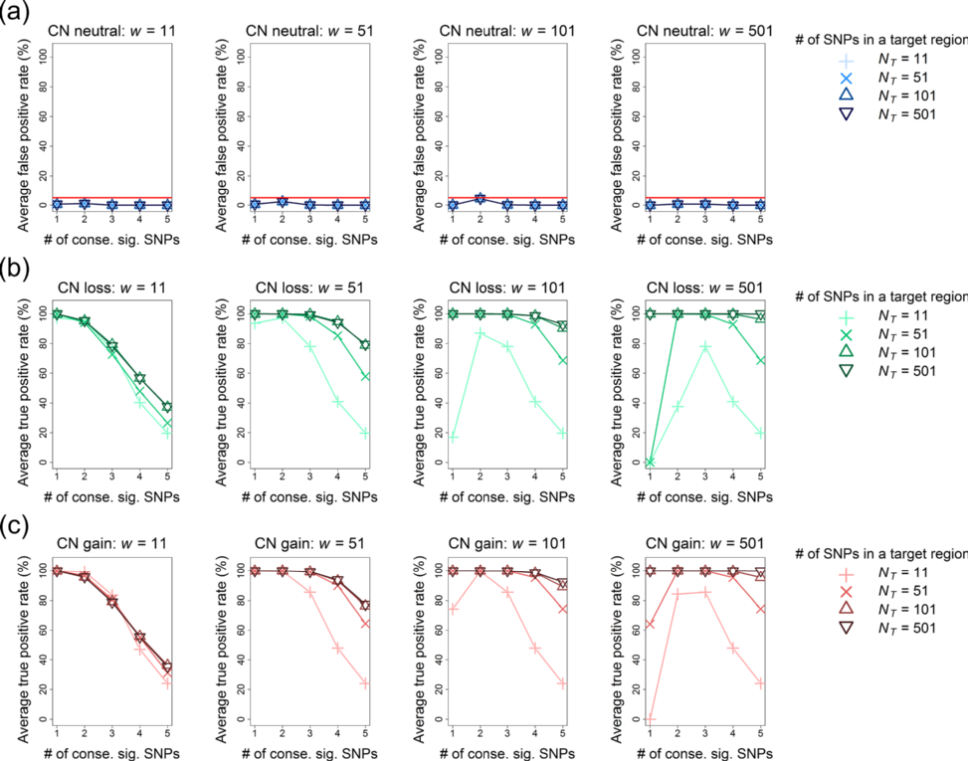
Average FPRs and TPRs of multi-point CNV/CNA analysis without noise interference (*q*% = 0 %). Panels from left to right show the results of four window sizes (*w* = 11, 51, 101, and 501) in order. In each subfigure, the symbols +, x, ∆, and ∇ indicate *N*_*T*_ = 11, 51, 101, and 501 SNPs in the target region, respectively. The horizontal axis indicates the setting of the number of consecutive significant SNPs (*n*_*c*_ = 1, 2, 3, 4, and 5). **a** The average FPRs (%) under the CN-neutral scenario. The horizontal reference line represents an FPR of 0.05. **b** The average TPRs (%) under the CN-loss scenario. **c** The average TPRs (%) under the CN-gain scenario

**Fig. 12:**
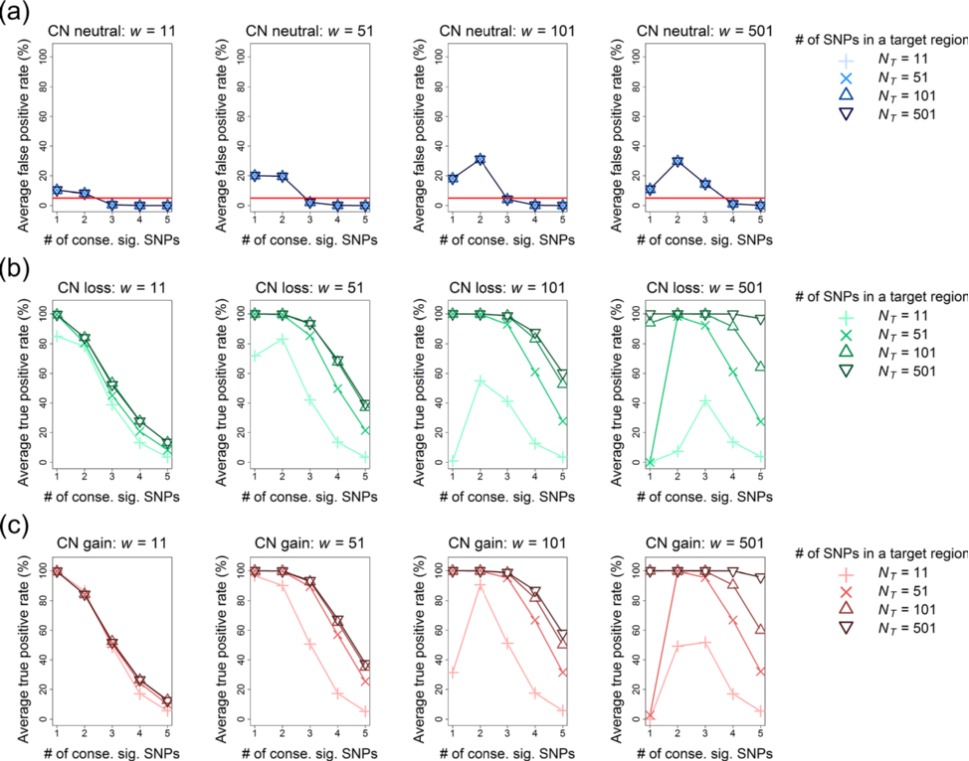
Average FPRs and TPRs of multi-point CNV/CNA analysis with noise interference (*q*% = 25 %). Panels from left to right show the results of four window sizes (*w* = 11, 51, 101, and 501) in order. In each subfigure, the symbols +, x, ∆, and ∇ indicate *N*_*T*_ = 11, 51, 101, and 501 SNPs in the target region, respectively. The horizontal axis indicates the setting of the number of consecutive significant SNPs (*n*_*c*_ = 1, 2, 3, 4, and 5). **a** The average FPRs (%) under the CN-neutral scenario. The horizontal reference line represents an FPR of 0.05. **b** The average TPRs (%) under the CN-loss scenario. **c** The average TPRs (%) under the CN-gain scenario

The results of region-level FPRs after a Bonferroni correction in the multipoint CNV/CNA analysis for *q*% = 0 % and 25 % are summarized in Figs. 11a and 12a, respectively. First, the FPRs inflated with an increase in the proportion of noise interference (*q*%). When the data was free of noise interference (i.e., *q*% = 0 %), the FPR was well controlled below a value of 0.05. However, the FPR became out of control when *q*% increased to 25 %. Second, a proper choice of window size (*w*) and the number of consecutive significant SNPs (*n*_*c*_) can reduce the FPR in CNV/CNA detection. When *q*% = 0 %, any *n*_*c*_ from 1 to 5 is favorable for *w* = 11, 51, 101, and 501 (Fig. 11a). When *q*% = 25 %, *n*_*c*_ ≥ 3 is recommended for *w* = 11, 51, and 101, and *n*_*c*_ ≥ 4 is recommended for *w* = 501 (Fig. 12a).

#### TPR of multipoint CN detection

The results of region-level TPRs in the multipoint CNV/CNA analysis after a Bonferroni correction are summarized: for *q*% = 0 %, the results are shown in Fig. 11b and c for CN loss and gain, respectively; *q*% = 25 %, the results are shown in Fig. 12b and c for CN loss and gain, respectively. First, an increasing proportion of noise interference (*q*%) reduced the TPR. Second, the TPR of the multipoint CNV/CNA analysis was higher than that of the single-point CNV/CNA analysis. Third, the TPR decreased with an increase in the required number of consecutive significant SNPs (*n*_*c*_). Exceptions occurred only when the window size (*w*) was significantly larger than the number of SNPs in the target region (*N*_*T*_). Finally, a larger *w* tended to increase TPR except for that *w* was significantly larger than *N*_*T*_. However, as mentioned in the previous subsection, an over-large window size increased the FPR, especially for the data with noise interference (Fig. 12a).

**Fig. 13:**
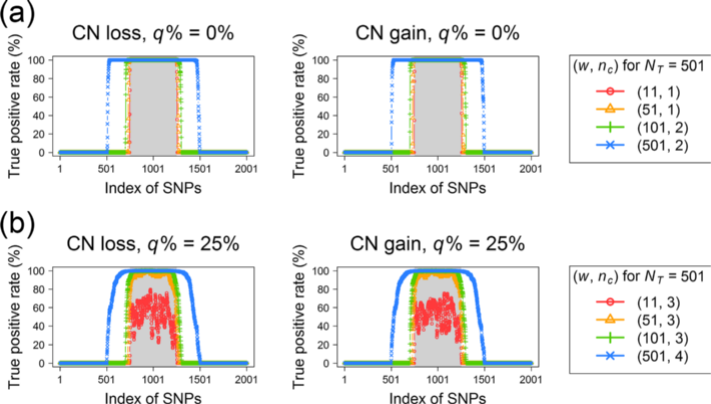
Accuracy of the detection region in the sliding-window multi-point CNV/CNA analysis. **a** Data without noise interference (*q*% = 0 %); **b** Data with noise interference (*q*% = 25 %). The left and right panels show the TPRs and FPRs for CN loss and CN gain, respectively. The target region of CN change is shaded on a gray background. The curves with red circles (*w* = 11), orange triangles (*w* = 51), green plus signs (*w* = 101), and sky-blue crosses (*w* = 501) represent the TPRs when the corresponding suboptimum settings of (*w*, *n*_*c*_) were used

We further evaluated the effect of *w* on the length of the identified chromosomal aberration. Under the premise of a well-controlled FPR, for each *w*, we selected a minimum *n*_*c*_, which attained the maximum TPR over different values of *N*_*T*_ as follows: for *q*% = 0 %, we recommend *n*_*c*_ = 1 or 2 for *w* = 11 and 51 and *n*_*c*_ = 2 for *w* = 101 and 501; and for *q*% = 25 %, we recommend *n*_*c*_ = 3 for *w* = 11, 51, and 101 and *n*_*c*_ = 4 for *w* = 501. The TPRs under the abovementioned suboptimal settings are summarized in Fig. 13a for *q*% = 0 % and Fig. 13b for *q*% = 25 %. The results suggest that although the use of a large *w* helped gain a high TPR, it led to an over-wide region of chromosomal aberration (green and blue curves in Fig. 13). By contrast, the use of small *w* not only reduced the TPR but also accurately located the region of chromosomal aberration (red and orange curves in Fig. 13).

Finally, we recommended the following optimal settings of (*w*, *n*_*c*_) for *N*_*T*_ = 11, 51, 101, and 501. For *q*% = 0 %, we recommend (*w*, *n*_*c*_) = (11, 2). The average FPR ranged from 1.12 to 1.12 %, and the average TPR ranged from 94.08 to 99.39 % (Table 1). For *q*% = 25 %, we recommend (*w*, *n*_*c*_) = (51, 3). The average FPR ranged from 2.19 to 2.19 %, and the average TPR ranged from 42.09 to 94.03 % (Table 1).

**Table 1:** Average FPRs and TPRs under the suggested settings of (*w*, *n*_*c*_) for the data without and with noise interference

Level of noise interference	Suggested setting^a^	FPR and TPR (Simulation scenario)	Number of SNPs in the target region			
	(*w*, *n*_*c*_)		*N*_*T*_ = 11	*N*_*T*_ = 51	*N*_*T*_ = 101	*N*_*T*_ = 501
Without noise interference (*q*% = 0 %)	(11, 2)	Average FPR (neutral)	1.12 %	1.12 %	1.12 %	1.12 %
		Average TPR (loss)	95.55 %	94.08 %	95.39 %	95.43 %
		Average TPR (gain)	99.39 %	96.69 %	95.59 %	95.83 %
With noise interference (*q*% = 25 %)	(51, 3)	Average FPR (neutral)	2.19 %	2.19 %	2.19 %	2.19 %
		Average TPR (loss)	42.09 %	85.45 %	94.03 %	93.54 %
		Average TPR (gain)	50.67 %	89.37 %	92.81 %	93.55 %

^a^ *w* Window size, *n*_c_ Number of consecutive significant SNPs, *FFRs* False positive rates, *TPRs* True positive rates, *N*_*T*_ Number of SNPs in the target region

### ALICE software

**Fig. 14:**
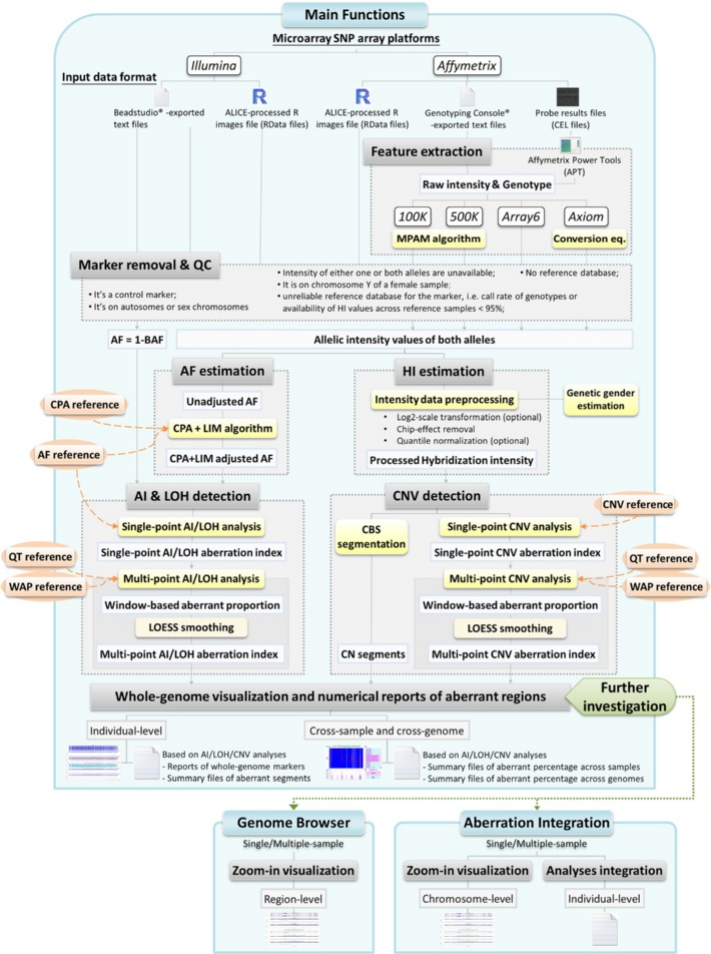
The structure of ALICE software

ALICE, programmed in R and R-GUI, is the software with a user-friendly interface for an integrated genomic analysis of AF, LOH/LCSH, AI, and CNV/CNA. The software, reference databases, library files for APT, annotation files, test examples, and user manual can be downloaded from the ALICE homepage (http://hcyang.stat.sinica.edu.tw/software/ALICE.html). ALICE consists of three main components—“Main Functions” (Additional file 11), “Genome Browser” (Additional file 12), and “Aberration Integration” (Additional file 13). The software structure is depicted in Fig. 14.

The first component “Main Functions” provides a whole-genome analysis of AF, LOH/LCSH, AI, and CNV/CNA. It has five functions, which are described in brief as follows:

Type of analysis: ALICE supports both the unpaired- and paired-sample analyses.

Input/output path: (1) Users should specify the directory in which they save the data to be analyzed. Moreover, users should download the annotation files, reference databases, and library files for Affymetrix Power Tools (APT) software (http://www.affymetrix.com/estore/partners_programs/programs/developer/tools/powertools.affx) from the ALICE homepage and save them in the input directory. (2) All numerical and graphical results and a log file will be saved automatically in the user-specified output directory.

Data format: (1) Users should specify a genotyping platform in their analysis. (2) Users can choose to provide (a) the Affymetrix CEL files, (b) Genotype/Intensity text files exported by the Affymetrix GTC or Illumina BeadStudio, or (c) the. RData files from their previous ALICE analysis.

Statistical analysis: (1) For intensity data preprocessing, users can choose to run (a) log_2_ transformation, (b) chip effect removal, or (c) quantile normalization. (2) For CNV/CNA segmentation, users can adjust five critical tuning parameters in the CBS algorithm [91, 92]: (a) significance level for the test of change point, (b) minimum number of markers in a segmentation region, (c) number of permutations for *p* value calculation in the test of change point, (d) the proportion of data trimming for removing outliers, and (e) cut-off for HI values of significant segments. In addition, uses can choose to use the original or quick CBS algorithm. (3) For AI/LOH/LCSH/CNV/CNA detection, users should specify the following: (a) the type of references (genotype-specific or nongenotype-specific) used in constructing the reference confidence interval, (b) the confidence level used in single-point and multipoint tests for AI/LOH/LCSH/CNV/CNA detection, (c) the window size and number of consecutive significant markers in genome scan, and (d) the upper bound level of references used in a multipoint test for AI/LOH/LCSH/CNV/CNA detection. According to the aforementioned user settings, a window will pop up to remind the users about which reference database should be saved in the input directory by clicking the “Run” button.

Output: (1) The numerical output includes: (a) raw.RData files of genotype and intensity data of all samples, (b) the APT output files, (c) the data description file about the parameter settings in the analysis, and (d) the output of an individual sample, which contains the results of single-point and multipoint detections for AI, LOH/LCSH, and CNV/CNA. In addition, consecutive significant markers identified by the single-point analysis or overlapping significant windows identified by the multipoint analysis will be joined and reported in the output. (2) The graphical output includes the following: (a) individual-sample plots and (b) cross-sample plots. In the individual-sample plots, users can select to draw a genome-wide AF and six-panel plots for each individual. The genome-wide six-panel plot includes the following: (i) the AF plot, (ii) the AI plot, (iii) the LOH/LCSH plot, (iv) the HI and CNV/CNA segmentation plot, (v) the CNV/CNA plot, and (vi) the *p* value plot of CNV/CNA detection (Additional file 1). In the cross-sample plot, users can select to draw the following: (i) the AI plot, (ii) the LOH/LCSH plot, and (iii) the CNV/CNA plot (see Additional file 14 as an example of the LOH/LCSH plot).

The second component “Genome Browser” provides a detailed visualization of the regions of AF, AI, LOH/LCSH, and CNV/CNA. First, users should specify the input and output directories specified in the previous ALICE analysis. They should then provide information on the group and ID of the samples of interest, the target genomic region, genetic markers (SNP or SNP + CNV/CNA), and the analysis plots comprising the following: (i) the AF plot, (ii) the AI plot, (iii) the LOH/LCSH plot, (iv) the CNV/CNA segmentation plot, (v) the CNV/CNA plot, and (vi) the *p* value plot of CNV/CNA detection. Genes located in the specified region of interest will be shown at the bottom of this interface. Moreover, the results of single-sample or multiple-sample visualization will be saved automatically to the prespecified output directory.

The final component “Aberration Integration” provides an integrated display of the numerical and graphical outputs of AF, AI, LOH/LCSH, and CNV/CNA. Similar to the procedures in the second component, users should select the input and output directories specified in the previous ALICE analysis and a single-sample or multiple-sample integration with the following settings: (1) genetic markers (SNP or SNP + CNV/CNA), (2) analysis method (single-point or multipoint analysis), (3) types of genomic aberrations, namely (a) AI + LOH/LCSH, (b) AI + CNV/CNA, (c) LOH/LCSH + CNV/CNA, and (d) AI + LOH/LCSH + CNV/CNA. The graphical and numerical outputs will be provided accordingly.

### ALICE genomic reference databases

We constructed the ALICE reference databases on the basis of the following conditions: (1) Ethnic group: Han Chinese population, other populations (African, Caucasian, and Asian populations), and the combined population; (2) SNP array: Affymetrix 100 K, 500 K, Array 6.0, Axiom CHB 1, and Illumina HumanHap 550 K; (3) Data format: CEL-based data, which is provided only for the Affymetrix platform, and genotype or intensity-based data for the Affymetrix (GTC) and Illumina (BeadStudio) platforms; (4) Window size and the number of consecutive significant markers: (*w*, *n*_*c*_) = (11, 2) and (51, 3); (5) Log_2_-scale transformation: the HI data with or without a log_2_-scale transformation; (6) Chip effect removal: mean or median; and (7) Quantile normalization: the HI data with or without an adjustment using a quantile normalization. A combination of three of the preprocessing conditions (i.e., the fifth, sixth, and seventh conditions) leads to eight databases, DB_1–DB_8, for each combination of ethnic groups, SNP arrays, data formats, window size, and the number of consecutive significant markers.

## Discussion

Axiom arrays, such as Axiom CHB 1, are widely used for conducting population-specific genomic studies. However, no CN probes have been designed for the Axiom arrays. Originally, Axiom arrays were developed for SNP genotyping, but not for CNV/CNA analysis. In this study, we developed ALICE for an integrated analysis of whole-genome HI and genotype data using Axiom arrays. The results of the real data analyses (Figs. 1, 2, 3, 4, 5, 6 and 7; Additional files 1, 2, 3, 4, 5, 6, 7, 8, 9 and 10), qPCR assays (Fig. 8), and simulation studies (Figs. 10, 11, 12 and 13) reveal that the Axiom array optimally performs the integrated analysis of AF, LOH/LCSH, AI, and CNVs/CNAs.

Although this paper mainly discusses the Affymetrix Axiom platform, ALICE also supports data analyses conducted using other Affymetrix SNP arrays, such as Array 6.0, and Illumina SNP arrays, such as Illumina 550 K. The analysis procedures for the non-Axiom platforms are detailed in the User Manual of ALICE: brief version (http://hcyang.stat.sinica.edu.tw/software/ALICE/Version1.0/Brief_Guide_for_Default_Example.pdf) and full version (http://hcyang.stat.sinica.edu.tw/software/ALICE/Version1.0/User%20Manual.pdf). Array 6.0 is another premier SNP array of Affymetrix, Inc., which has been broadly used in genomic studies. The main differences between Axiom and Array 6.0 are as follows: (1) DNA preparation (the total amount of genomic DNA and range of DNA fragments), (2) probe design (ligation, probe sequence, probe length, and the number of replicates of probe pairs), (3) marker contents (the number of SNPs, inclusion of CN probe, and source databases), and (4) genotyping experiment (inclusion of control sample, number of samples in a chip, and experimental cost).

First, regarding DNA preparation, Axiom requires 100–200 ng of genomic DNA, which is less than that required by Array 6.0, 500 ng. After restriction enzyme digestion, the DNA fragments of Axiom (25–125 base pairs) are shorter than those of Array 6.0 (200–1,100 base pairs). Second, regarding probe design, Axiom uses on-chip and solution probes that are linked together by ligating if they perfectly match the same target DNA, whereas Array 6.0 uses only on-chip probes that hybridize to the target DNA. In Axiom, only one on-chip probe is designed for detecting non-C/G and non-A/T polymorphisms; however, two on-chip probes are designed for detecting C/G and A/T polymorphisms. In Array 6.0, two probes are designed that are complementary to an individual allele of a SNP. The lengths of Axiom on-chip and solution probes are 30 and 9 mer, respectively, while that of an Array 6.0 probe is 25 mer. Axiom uses two-color labeling of solution probes to distinguish SNP alleles, whereas Array 6.0 uses only single-color labeling of target DNA for detecting signals. Axiom designs two replicates of probe pairs, and Array 6.0 designs 3–4 replicates.

Third, regarding marker contents, Axiom provides approximately 640,000 SNPs, whereas Array 6.0 provides approximately 906,600 SNPs and 946,000 CN probes. Axiom collects markers from the Axiom Genomic Databases (http://www.affymetrix.com/support/technical/sample_data/axiom_db/axiomdb_data.affx), which contain web-validated and fully annotated SNPs in the International HapMap Project [43, 65, 66], 1000 Genomes Project [42, 45], and dbSNP database [67]; Array 6.0 collects markers from the International HapMap Project and the Database of Genomic Variants [93]. Finally, regarding the genotyping experiment, Axiom includes one control sample in a plate and simultaneously genotypes 96 or 384 samples in the same plate; Array 6.0 genotypes each sample or chip individually. The genotyping cost of Axiom is much lower than that of Array 6.0. The average genotyping cost for one sample is approximately 3,400 NTD for Axiom and 13,400 NTD for Array 6.0 in the National Center for Genome Medicine (http://ncgm.sinica.edu.tw/affymetrix_user_01.html).

The longer probe sequences and two-color ligation designs in Axiom compared with those in Array 6.0 have increased the sensitivity and specificity of signal detection and ability to differentiate between the HI measures of two alleles. In addition, the higher flexibility in designing SNP contents and lower experimental cost allow researchers to obtain a content-optimum solution and perform a cost-effective experiment for a population-specific genomic study. These advantages have attracted numerous researchers to conduct large-scale genetic or genomic projects using Axiom arrays [77–79, 81, 94–96]. Lu et al. conducted a genome-wide association study on coronary artery disease-genotyped 1,034 patients and 4,245 controls using Axiom CHB 1 arrays [77]. Shi et al. conducted a genome-wide association study on cervical cancer and genotyped 1,364 patients and 3,028 controls using Axiom CHB 1 arrays [78]. In addition, we are conducting the Taiwan Biobank Project in Taiwan, which aims to collect 200,000 normal controls and 100,000 disease patients (http://www.twbiobank.org.tw/). All the samples will be genotyped using the Axiom TWB arrays, which we designed for the Taiwan population. The analysis functions and software developed in this study will significantly enhance the utilities of Axiom arrays in medical and population genomics.

We developed single-point and multipoint statistical methods on the basis of confidence intervals and hypothesis testing for detecting the regions of CNVs/CNAs. Our methods incorporate information on AI and LOH/LCSH into the CNV/CNA analysis to increase the accuracy and efficiency of adjusted HI data, CN segmentation, and CNV/CNA detection. This new implementation was not considered in previous CNV/CNA analyses. First, the method uses information on AI for processing HI data. After scale normalization, chip effect removal, and quantile normalization, the influence of aberrant-probe perturbation was adjusted. The HI values of all SNPs in a sample are subtracted from the average HI of non-AI SNPs. The AI SNPs must be excluded from the calculation of the average because their HI values are interfered by chromosomal aberrations [6] or poor data quality [97]. After normalization, the HI values of different samples become comparable. A zero value, large positive value, and large negative value of HI reflect the statuses “no CNV/CNA,” “CN gain,” and “CN loss,” respectively.

In addition, we developed an efficient segmentation method for detecting the range of CNVs/CNAs. Studies have reported that CBS can detect CNVs/CNAs, accurately determine their boundaries, and optimally control the FPR [98–100]. However, CBS has two major limitations [98–101]. First, CBS requires a very intensive computation. Second, CBS does not use allelic information, because CBS was originally developed for a-CGH data segmentation. To overcome the two limitations, we developed a quick CBS algorithm to enhance the performance of CBS. The new algorithm incorporates AI, LOH/LCSH, and allelic information and assigns higher weights to the SNPs that exhibit a stronger signal for chromosomal aberrations. Several real data analyses reveal that compared with the original CBS algorithm, the quick CBS algorithm can provide reasonable results and save approximately 30 % and 67 % of computational time in the analyses of cancer and noncancerous samples, respectively.

We also evaluated the performance of our CNV/CNA detections by conducting simulation studies. For the whole-genome single-point CNV/CNA detection, adjustments for multiple tests such as Bonferroni corrections are required. The simulation results indicated that the FPR was optimally controlled. However, the TPR was low when the effect size was small (Fig. 10). Thus, we developed the multipoint methods for increasing the TPR of the single-point method. In the multipoint methods, a chromosome is partitioned using overlapping sliding windows, and SNP markers in the same sliding window are integrated to detect CNVs/CNAs. The simulation results indicated that window size and the number of consecutive significant SNPs are the two main factors affecting the FPR and TPR. In general, using a large window increases the TPR; however, it also increases the FPR and overestimates the lengths of the regions of CNVs/CNAs (Figs. 11, 12 and 13). Using a larger number of consecutive significant SNPs reduces the FPR; however, it also reduces the TPR (Figs. 11 and 12). We therefore recommend two optimal settings for the scenarios with and without noise interference on the basis of the results in Figs. 11, 12 and 13. However, the users are still recommended to attempt more window sizes according to the features in their own studies. The analysis can be initiated using a slightly larger window to identify a relatively large region of chromosomal aberrations. Thereafter, smaller windows can be used gradually to finely map the exact region of chromosomal aberrations and determine the accurate boundaries of chromosomal aberrations.

Our method considers several procedures to avoid potential false positive findings. First, ALICE uses a Bonferroni correction to adjust for the multiple-testing problem. Second, ALICE requires a detected CNV/CNA to satisfy the following criteria: (1) its window-based aberrant proportion (WAP) value should be higher than that from a reference database and (2) its Bonferroni-corrected *p* value should be significant in the multipoint analysis. Third, ALICE can control consecutive significant SNPs in a CNV/CNA. In addition, the real data analysis of admixed samples indicated that AI and LOH/LCSH may be the precursors of CNVs/CNAs. A region of CNVs/CNAs is more reliable if AI and LOH/LCSH are also detected in the same region. Finally, the CBS analysis can aid in detecting CNVs/CNAs and determining the boundaries of the regions of CNVs/CNAs. The results of our simulation and real data analysis revealed that the FPR can be optimally controlled.

Recently, Affymetrix, Inc. launched the Axiom CNV Summary Tool (http://www.affymetrix.com/catalog/prod820008/AFFY/Axiom%26%23174%3B+CNV+Summary+Tools+Software#1_1) and cooperated with commercial software, Nexus Copy Number software, from BioDiscovery, Inc. to detect CNVs/CNAs. The performance of the commercial tool for the Axiom array data was not evaluated using rigorous simulation studies. The method was not developed according to the data characteristics of Axiom and cannot use population-specific genomic reference databases, which are crucial for genomic studies. In addition, the annual software expense may increase budget loading and limit the broad use. ALICE is the first freeware that offers an integrated analysis of AF, AI, and LOH/LCSH and the detection of CNVs/CNAs for the Axiom platform on the basis of genotype and HI data.

In ALICE, the detection of AI, LOH/LCSH, and CNVs/CNAs relies on genomic reference databases. We have completed the ALICE databases for Axiom CHB 1. Currently, we are constructing the databases for Axiom CHB 2, TWB, and other populations using data from public databases such as Gene Expression Omnibus [102] and the Database of Genotypes and Phenotypes [103]. These resources will become available on the ALICE homepage in the near future.

## Conclusions

The Affymetrix Axiom SNP arrays provide a high-density and high-throughput genotyping solution for a population-optimized analysis at a lower cost. However, there was no free software available for an integrated analysis of AF, AI, LOH/LCSH, and CNV/CNA. In this paper, we introduce the ALICE software developed for Axiom and other SNP arrays. ALICE consists of the CPA + LIM method for an AF adjustment, the single-point and multipoint methods for an integrated analysis of AI, LOH/LCSH, and CNV/CNA detection, and the CBS algorithms for CNV/CNA segmentation. In addition, ALICE is featured by a user-friendly interface that provides several genomic reference databases and a convenient genomic browser for visualizing the analysis results. The accuracy, reliability, and efficiency of ALICE have been carefully evaluated by (1) the simulation studies, (2) real data analyses of genomic data sets of normal samples, cancer cell samples, and admixed samples of cancer cell lines and the corresponding blood cell line, and (3) validation using qPCR assays. We believed that ALICE will provide a powerful statistical and bioinformatics tool for analyzing the modern SNP arrays in medical and population genomics.

## Methods

### Sample materials and genotyping

In this study, 3,236 unrelated samples were collected from the following three sources: (1) 3,025 from Taiwan Han Chinese Cell and Genome Bank [104], (2) 210 from the International HapMap Project II [66], and (3) one from a metastatic small-cell lung cancer cell line and the corresponding blood cell line from American Type Culture Collection (http://www.atcc.org/). All the samples were genotyped using at least one of the following Affymetrix gene chips: the Affymetrix Human Mapping 100 K Array Set, Affymetrix Human Mapping 500 K Array Set, Affymetrix Genome-Wide Human SNP Array 6.0, Affymetrix Axiom Genome-Wide CHB 1 Array Plate (Affymetrix, San Diego, CA, USA), and Illumina HumanHap550-Duo v3 Genotyping BeadChip (Illumina Inc., San Diego, CA, USA). For brevity, we have used “Affymetrix 100 K,” “500 K,” “Axiom,” “Array 6.0,” and “Illumina 550 K” throughout this paper. The data sheets and genotyping manuals for these SNP chips can be downloaded from the Technical Documentation Download web page on the Affymetrix (http://www.affymetrix.com/support/technical/index.affx) and Illumina websites (http://www.illumina.com/). All the participants involved in the genomic projects signed informed consent forms.

For the first source, we included the arrays that passed a genotyping quality examination performed using SAQC software [97] and had no apparent chromosomal aberrations. In total, 367, 448, 1,013, 1,666, and 854 samples were genotyped using Affymetrix 100 K, 500 K, Axiom, Array 6.0, and Illumina 550 K, respectively. All these samples were used for constructing the ALICE genomic reference databases of our own population of Taiwan for different genotyping platforms. The 1,666 samples genotyped using the Axiom arrays were also used in a simulation study for a TPR and FPR analysis. In addition to the normal unrelated samples, we analyzed 15 extra samples, which were genotyped using both Axiom and Array 6.0 and exhibited apparent chromosomal aberrations, for comparing the results between the Axiom and Array 6.0 platforms.

For the second source, we studied 210 unrelated samples, which consisted of the following: (1) 60 African-descendant Yoruba samples from Ibadan, Nigeria, (2) 60 European-descendant samples from CEPH Utah residents in USA, and (3) 90 East-Asian-descendant samples, comprising 45 Han Chinese in Beijing and 45 Japanese in Tokyo. All the samples were genotyped using Affymetrix 100 K, 500 K, and Array 6.0. Their genotypes, intensities, and CEL files were downloaded from the HapMap website (http://hapmap.ncbi.nlm.nih.gov/). All the samples were used for constructing the ALICE genomic reference databases of African, European, and Asian populations for different genotyping platforms.

For the final source, a metastatic small-cell lung cancer cell line (NCI-H2171) and the corresponding blood cell line (NCI-BL2171) were purchased from ATCC. NCI-H2171 is a hypodiploid cell line that carries multiple chromosomal deletions and duplications. The genomic DNA of the cancer cell line (*p*%) was mixed with that of the blood cell line [(100 - *p*)%] in an admixture experiment. The admixture proportion *p*% ranged from 0 to 100 %, with increments of 10 %. All the 11 samples were duplicated and genotyped using Axiom, whereas the pure cancer cell line and blood cell line were genotyped using Array 6.0. The 11 admixed samples were used to evaluate the effect of admixture proportion on unpaired- and paired-sample integrated analyses of AF, AI, LOH/LCSH, and CNV/CNA and on the real-time qPCR-based validation experiments.

### Ethics, consent and permissions

All the participants involved in the genomic projects signed informed consent forms. This study was approved by Human Subjects Research Ethics, Academia Sinica [AS-IRB01-11077 (08039)].

### Extraction of HI

Self-developed R functions in ALICE were used to prepare the HI, genotype, and annotation data for different SNP array platforms. For the Affymetrix Axiom platform, ALICE can read data from two types of data files, the probe result files (*.CEL) and the “log2 ratio and strength data” files (*.TXT). In the case of the probe result files, ALICE generates batch codes (*.BAT) to call the “--summaries” command in the “apt-probeset-genotype” module of the APT software. Then, the whole-genome allele-specific HI and genotype data of all individuals is saved in the files AxiomGT1.summary.txt and AxiomGT1.calls.txt, respectively. The file sizes can be too large to read if the number of study individuals is large. To overcome the bottleneck in reading the large amount of data, ALICE uses the filehash package in R language to dump the files into a hard drive. Finally, for each individual, the HI, genotype, and annotation information is combined and saved in an R workspace file (*.RData) for downstream data analysis.

In the case of the “log2 ratio and strength data” files (*.TXT) generated from the Affymetrix Genotyping Console™ (GTC) software (http://www.affymetrix.com/estore/browse/level_seven_software_products_only.jsp?productId=131535#1_1) by each individual, ALICE converts the log ratio and strength values into the allele-specific HI values of two alleles as follows: Let *S* denote the arithmetic mean of the log_2_-scale HI values of two alleles (i.e., “Strength”) and *L* denote a log_2_-scale ratio of the HI of allele *A* to that of allele *B* (i.e., “Log Ratio”). The *S* and *L* data of every SNP are provided in a *.TXT file. ALICE solves a system of two mathematical equations to derive the HI values of alleles *A* and *B*, that is, (*h*_*A*_,*h*_*B*_), as follows: *h*_*A*_ = 2^*S* + 0.5*L*^ and *h*_*B*_ = 2^*S* − 0.5*L*^. For each individual, the HI, genotype, and annotation data are saved in an R workspace file (*. RData) for the downstream data analysis. The extraction of HI values for other SNP array platforms is detailed in the User Manual of ALICE.

### Preprocessing of HI

HI reflects a relative CN. For a SNP, the total HI is obtained by summing the HI values of two alleles. In addition to SNP probes, several SNP array platforms provide CN probes for measuring the CN. For example, Affymetrix Array 6.0 simultaneously provides more than 906,600 SNP and 946,000 CN probes. For a CN probe, the HI is measured by averaging the HI values of the replicates of the same CN probes. We used several optional data preprocessing procedures to normalize HI values, namely: (1) scale normalization by taking a log_2_ transformation; (2) chip effect removal by subtracting the mean or median from the HI and then dividing by the standard deviation of HI of all SNPs in the same array; (3) quantile normalization for removing technical variation in the probes [105]; and (4) removal of aberrant-probe perturbation as follows: Suppose that there are *M* autosomal SNPs. Let *s*_*i*_ denote the HI value of the *i*^th^ autosomal SNP probe after the first three steps. Event Δ_*i*_ indicates that the *i*^th^ autosomal SNP is in a status of allelic balance (refer to the Background section for the definition of allelic balance). The final HI value of the *m*^th^ autosomal SNP probe is calculated as follows:

$$ {t}_m={s}_m - \frac{{\displaystyle {\sum}_{i=1}^M}{s}_i\cdot I\left[{\Delta}_i\right]}{{\displaystyle {\sum}_{i=1}^M}I\left[{\Delta}_i\right]}, $$

where the indicator function *I*[Δ_*i*_] equals unity if the event Δ_*i*_ is held; otherwise, it equals zero.

ALICE detects chromosomal abnormalities by examining the patterns of AF, AI, and LOH/LCSH on the basis of the raw HI data and those of CNVs/CNAs on the basis of the final HI data. The graphical results are summarized in a six-panel figure: AF plot, AI plot, LOH/LCSH plot, HI and CN segmentation plot, CNV/CNA plot, and *p* value plot (Additional file 1). The following subsections describe the statistical methods used in each of the six subfigures.

### Individual-level AF estimation with a CPA + LIM adjustment

AFs are of two types: population-level AF and individual-level AF [4, 6]. This paper focuses on the individual-level AF; therefore, throughout this paper, we have omitted the word “individual-level” when discussing AF. ALICE estimates an AF using a two-step procedure that integrates the CPA [106] and LIM [107]. Let *h*_*i*,*m*_ denote the relative HI of allele *A* of the *m*^th^ SNP of the *i*^th^ individual and *n*_*m*_(*g*) denote the number of individuals who had genotype *g* on the *m*^th^ autosomal SNP in the normal reference samples, where genotype *g* is *AA*, *AB*, or *BB*. In the first step, we estimate the AF of allele *A* of the *m*^th^ autosomal SNP with an adjustment for CPA as follows:

1 $$ {\hat{h}}_{i,m}=\frac{h_{i,m}}{h_{i,m}+{\kappa}_m\cdot \left(1-{h}_{i,m}\right)} $$

where the CPA is calculated as follows [23, 106]:

$$ {\kappa}_m=\frac{1}{n_m(AB)}{\displaystyle {\sum}_{i=1}^{n_m(AB)}\frac{h_{i,m}}{1-{h}_{i,m}}+\frac{n_m(AB)}{n_m(AB)-1}}\times \left[\frac{h_{i,m}}{1-{h}_{i,m}}-\frac{1}{n_m(AB)}{\displaystyle {\sum}_{i=1}^{n_m(AB)}\frac{h_{i,m}}{1-{h}_{i,m}}}\right]. $$

In the second step, we further calibrate the AF using the LIM. Across the normal reference samples, the average of the genotype-specific AFs for genotype *g* on the *m*^th^ autosomal SNP is written as follows:

$$ {\overline{h}}_{+,m}(g)=\frac{1}{n_m(g)}{\displaystyle {\sum}_{i=1}^{n_m(g)}{\hat{h}}_{i,m}(g)}. $$

The CPA + LIM AF is calculated as follows:

$$ {\hat{f}}_{i,m}=\left\{\begin{array}{cc}\hfill 1,\hfill & \hfill \mathrm{if}\ {\overline{h}}_{+,m}(AA)<{\hat{h}}_{i,m}\hfill \\ {}\hfill \frac{1}{2}+\frac{1}{2}\cdot \frac{{\hat{h}}_{i,m}-{\overline{h}}_{+,m}(AB)}{{\overline{h}}_{+,m}(AA)-{\overline{h}}_{+,m}(AB)},\hfill & \hfill \mathrm{if}\kern0.75em {\overline{h}}_{+,m}(AB)<{\hat{h}}_{i,m}\le {\overline{h}}_{+,m}(AA)\hfill \\ {}\hfill \frac{1}{2}\cdot \frac{{\hat{h}}_{i,m}-{\overline{h}}_{+,m}(BB)}{{\overline{h}}_{+,m}(AB)-{\overline{h}}_{+,m}(BB)},\hfill & \hfill \mathrm{if}\ {\overline{h}}_{+,m}(AB)<{\hat{h}}_{i,m}\le {\overline{h}}_{+,m}(BB)\hfill \\ {}\hfill 0,\hfill & \hfill \mathrm{if}{\hat{h}}_{i,m}\le {\overline{h}}_{+,m}(BB)\hfill \end{array}\right.. $$

#### Single-point index of AI detection

On the basis of the CPA + LIM AF estimates, ALICE incorporates our previous confidence interval approach [6] to detect AI for a SNP. Let ${\overline{f}}_{+,m}(g)=\frac{1}{n_m(g)}{\displaystyle {\sum}_{i=1}^{n_m(g)}{\widehat{f}}_{i,m}}\kern0.5em \mathrm{and}\kern0.5em {S}_{+,m}(g)={\left[\frac{1}{n_m(g)-1}{\displaystyle {\sum}_{i=1}^{n_m(g)}{\left({\hat{f}}_{i,m}-{\overline{f}}_{+,m}(g)\right)}^2}\right]}^{1/2}$denote the genotype-specific mean and standard deviation of the CPA + LIM AF estimates of the *m*^th^ autosomal SNP in the normal reference samples, respectively. Notation $\mathcal{A}$. stands for an AI analysis and *SP* stands for a single-point analysis. In an unpaired-sample analysis, the confidence intervals of AF of the *m*^th^ autosomal SNP for genotypes *AA*, *AB*, and *BB* can be constructed as follows:

2 $$ \left\{\begin{array}{l}C{I}_{+,m}^{\mathcal{A},SP}(AA)=\left[{\overline{f}}_{+,m}(AA)-{Z}_{1-\frac{\alpha }{3M}}\cdot {S}_{+,m}(AA),1\right]\hfill \\ {}C{I}_{+,m}^{\mathcal{A},SP}(AB)=\left[{\overline{f}}_{+,m}(AB) - {Z}_{1-\frac{\alpha }{6M}}\cdot {S}_{+,m}(AB),\ {\overline{f}}_{+,m}(AB) + {Z}_{1-\frac{\alpha }{6M}}\cdot {S}_{+,m}(AB)\right]\hfill \\ {}C{I}_{+,m}^{\mathcal{A},SP}(BB)=\left[0,\ {\overline{f}}_{+,m}(BB) + {Z}_{1-\frac{\alpha }{3M}}\cdot {S}_{+,m}(BB)\right]\hfill \end{array}\right. $$

where *Z*_*α*_ indicates the *α* quantile of a standard normal distribution and *M* is the number of SNPs on the chromosome where the *m*^th^ SNP is located. Our single-point AI is defined as follows:

$$ {I}_{i,m}^{\mathcal{A},SP}=\left\{\begin{array}{cc}\hfill 1,\hfill & \hfill \mathrm{if}\ {\hat{f}}_{i,m}\notin\ C{I}_{+,m}^{\mathcal{A},SP}(AA),\ C{I}_{+,m}^{\mathcal{A},SP}(AB),\ \mathrm{or}\ C{I}_{+,m}^{\mathcal{A},SP}(BB)\hfill \\ {}\hfill 0,\hfill & \hfill \mathrm{otherwise}\hfill \end{array}.\right. $$

Thus, the *m*^th^ SNP of the *i*^th^ individual is classified as AI if ${I}_{i,m}^{\mathcal{A},SP}=1$; otherwise, it is a non-AI SNP.

In a paired-sample analysis, the norms of normal reference are changed from the independent normal reference samples to the paired-normal tissue samples. The AI detector can be derived by replacing the sample mean and standard deviation of the CPA + LIM AF estimates in Equation (2) by the AF estimate and standard deviation of the paired-normal tissue sample. Because a paired-sample analysis considers only one of the three confidence intervals in Equation (2) according to the genotype of the SNP in the paired-normal tissue sample, *Z*_1 − *α*/3*M*_, *Z*_1 − *α*/6*M*_, and *Z*_1 − *α*/3*M*_ should be replaced by *Z*_1 − *α*/*M*_, *Z*_1 − *α*/2*M*_, and *Z*_1 − *α*/*M*_ in the three confidence intervals (i.e., $C{I}_{+,m}^{\mathcal{A},SP}(AA)$, $C{I}_{+,m}^{\mathcal{A},SP}(AB)$, and $C{I}_{+,m}^{\mathcal{A},SP}(BB)$) in Equation (2), respectively.

### Single-point index of LOH/LCSH detection

ALICE incorporates the confidence interval approach [6] to detect the status of LOH and LCSH. Notation ℒ stands for an LOH/LCSH analysis. In an unpaired-sample analysis, the confidence interval of LOH/LCSH of the *m*^th^ autosomal SNP can be constructed as follows:

3 $$ C{I}_{+,m}^{\mathrm{\mathcal{L}},SP}(AB)=\left[{\overline{f}}_{+,m}(AB) - {Z}_{1-\frac{\alpha }{2M}}\cdot {S}_{+,m}(AB),\ {\overline{f}}_{+,m}(AB) + {Z}_{1-\frac{\alpha }{2M}}\cdot {S}_{+,m}(AB)\right]. $$

Our single-point LOH/LCSH detector is defined as follows:

$$ {I}_{i,m}^{\mathrm{\mathcal{L}},SP}(AB)=\left\{\begin{array}{cc}\hfill 1,\hfill & \hfill \mathrm{if}\ {\hat{f}}_{i,m}\notin\ C{I}_{+,m}^{\mathrm{\mathcal{L}},SP}(AB)\hfill \\ {}\hfill 0,\hfill & \hfill \mathrm{otherwise}\hfill \end{array}\right.. $$

Thus, the *m*^th^ SNP of the *i*^th^ individual is classified as LOH/LCSH if *I*_*i*,*m*_^ℒ,*SP*^(*AB*) = 1; otherwise, it is a non-LOH/LCSH SNP.

In a paired-sample analysis, the norms of normal reference are changed from the independent normal reference samples to the paired-normal tissue samples. The LOH/LCSH detector can be derived by replacing the sample mean and standard deviation of the CPA + LIM AF estimates in Equation (3) by the AF estimate and standard deviation of the paired-normal tissue sample.

### Single-point index of CNV/CNA detection

ALICE considers the following two types of confidence interval approaches for detecting CN gain and loss: genotype-specific and nongenotype-specific methods. First, we discuss an unpaired-sample analysis based on the genotype-specific method. Let ${\overline{t}}_{+,m}(g)=\frac{1}{n_m(g)}{\displaystyle {\sum}_{i=1}^{n_m(g)}{t}_{i,m}}\kern0.5em \mathrm{and}\kern0.5em {\widehat{\sigma}}_{+,m}(g)={\left[\frac{1}{n_m(g)-1}{\displaystyle {\sum}_{i=1}^{n_m(g)}{\left({t}_{i,m}-{\overline{t}}_{+,m}(g)\right)}^2}\right]}^{1/2}$denote the genotype-specific mean and standard deviation of hybridization intensities of the *m*^th^ autosomal SNP in the normal reference samples, respectively. Notation $\mathcal{C}$ stands for a CNV/CNA analysis. The genotype-specific confidence intervals of CNVs/CNAs of the *m*^th^ autosomal SNP for genotypes *AA*, *AB*, and *BB* can be constructed as follows:

4 $$ C{I}_{+,m}^{\mathcal{C},SP}(g)=\left[{\overline{t}}_{+,m}(g)-{Z}_{1-\frac{\alpha }{2M}} \cdot {\hat{\sigma}}_{+,m}(g),\ {\overline{t}}_{+,m}(g)+{Z}_{1-\frac{\alpha }{2M}}\cdot {\hat{\sigma}}_{+,m}(g)\right]. $$

Our single-point genotype-specific CNV/CNA detector is defined as follows:

$$ {I}_{i,m}^{\mathcal{C},SP}(g)=\left\{\begin{array}{cc}\hfill 1,\hfill & \hfill \mathrm{if}\ {t}_{i,m} > {\overline{t}}_{+,m}(g)+{Z}_{1-\frac{\alpha }{2M}}\cdot {\hat{\sigma}}_{+,m}(g)\hfill \\ {}\hfill -1,\hfill & \hfill \mathrm{if}\ {t}_{i,m} < {\overline{t}}_{+,m}(g)-{Z}_{1-\frac{\alpha }{2M}}\cdot {\hat{\sigma}}_{+,m}(g)\hfill \\ {}\hfill 0\hfill & \hfill \mathrm{otherwise}\hfill \end{array}\right.. $$

Thus, the *m*^th^ SNP of the *i*^th^ individual is classified as a CN gain if ${I}_{i,m}^{\mathcal{C},SP}(g)=1$ and as a CN loss if ${I}_{i,m}^{\mathcal{C},SP}(g)=-1$; otherwise, this SNP has no CNVs/CNAs. The adjusted *p* value for the statistical test of CNVs/CNAs after the Bonferroni correction can be calculated as follows:

$$ {p}_{i,m}^{\mathcal{C},SP}= \min \left\{2\left(1-\Phi \left({Z}_{i,m}^{\mathcal{C},SP}\right)\right)\cdot M,1\right\}, $$

where ${Z}_{i,m}^{\mathcal{C},SP}=\left({t}_{i,m}-{\overline{t}}_{+,m}(g)\right)/{\hat{\sigma}}_{+,m}(g)$ is the test statistic and Φ( ⋅ ) is the cumulative distribution function of standard normal distribution.

Second, we discuss an unpaired-sample analysis based on the nongenotype-specific method. Let ${\overline{t}}_{+,m}=\frac{1}{n_m}{\displaystyle {\sum}_{i=1}^{n_m}{t}_{i,m}}\kern0.5em \mathrm{and}\kern0.5em {\hat{\sigma}}_{+,m}={\left[\frac{1}{n_m-1}{\displaystyle {\sum}_{i=1}^{n_m}{\left({t}_{i,m}-{\overline{t}}_{+,m}\right)}^2}\right]}^{1/2}$ denote the nongenotype-specific mean and standard deviation of hybridization intensities of the *m*^th^ autosomal SNP in the normal reference samples, respectively. The nongenotype-specific confidence intervals of CNVs/CNAs of the *m*^th^ autosomal SNP can be constructed as follows:

5 $$ C{I}_{+,m}^{\mathcal{C},SP}=\left[{\overline{t}}_{+,m}-{Z}_{1-\frac{\alpha }{2M}}\cdot {\hat{\sigma}}_{+,m},\ {\overline{t}}_{+,m}+{Z}_{1-\frac{\alpha }{2M}}\cdot {\hat{\sigma}}_{+,m}\right]. $$

Our single-point nongenotype-specific CNV/CNA detector is defined as follows:

$$ {I}_{i,m}^{\mathcal{C},SP}=\left\{\begin{array}{cc}\hfill 1,\hfill & \hfill \mathrm{if}\ {t}_{i,m} > {\overline{t}}_{+,m}+{Z}_{1-\frac{\alpha }{2M}}\cdot {\hat{\sigma}}_{+,m}\hfill \\ {}\hfill -1,\hfill & \hfill \mathrm{if}\ {t}_{i,m} < {\overline{t}}_{+,m}-{Z}_{1-\frac{\alpha }{2M}}\cdot {\hat{\sigma}}_{+,m}\hfill \\ {}\hfill 0,\hfill & \hfill \mathrm{otherwise}\hfill \end{array}.\right. $$

Thus, the *m*^th^ SNP of the *i*^th^ individual is classified as a CN gain if ${I}_{i,m}^{\mathcal{C},SP}=1$ and as a CN loss if ${I}_{i,m}^{\mathcal{C},SP}=-1$; otherwise, this SNP has no CNVs/CNAs. The adjusted *p* value for the statistical test of CNVs/CNAs after the Bonferroni correction can be calculated as follows:

$$ {p}_{i,m}^{\mathcal{C},SP}= \min \left\{2\left(1-\Phi \left({Z}_{i,m}^{\mathcal{C},SP}\right)\right)\cdot M,1\right\}, $$

where ${Z}_{i,m}^{\mathcal{C},SP}=\left({t}_{i,m}-{\overline{t}}_{+,m}\right)/{\hat{\sigma}}_{+,m}$ is the test statistic.

Furthermore, in a paired-sample analysis, the norms of normal reference are changed from the independent normal reference samples to the paired-normal tissue samples. The CNV/CNA detector can be derived by replacing the sample mean and standard deviation of hybridization intensities by the mean and standard deviation of hybridization intensities of the paired-normal tissue sample in Equation (4) for the genotype-specific method and in Equation (5) for the nongenotype-specific method.

### Multipoint indices of AI, LOH/LCSH, and CNV/CNA detection

A sliding-window multipoint approach was used to scan chromosomes to detect chromosomal aberrations as follows: First, all SNPs were ordered according to their physical positions on a chromosome. If a SNP array also provides CN probes, they are included but analyzed separately. Let an anchor denote a marker of interest and be located in the middle of a window. A window was constructed by collecting the anchor marker and its flanking markers; here, we considered an equal number of markers from the upstream and downstream of the anchor. Regarding the notation, a window anchored at the *m*^th^ marker with a size of *w* = 2*v* + 1 is denoted as *W*(*m*, 2*v* + 1) = {*m* − *v*, ⋯ *m* − 1, *m*, *m* + 1, ⋯, *m* + *v*}. The anchor was sequentially shifted from the starting marker to the ending one until all markers on a chromosome were scanned completely. Let $\mathrm{\mathcal{E}}=\mathcal{A}$, ℒ, or $\mathcal{C}$ denote AI, LOH/LCSH, or CNV/CNA, respectively. Notation *MP* denotes a multipoint analysis. We define our sliding-window multipoint statistic, which is a WAP, as follows:

$$ {W}_{i,m}^{\mathrm{\mathcal{E}},MP}\left(v,{n}_c\right)=\frac{1}{2v+1}{\displaystyle {\sum}_{x\in \left\{m-v,m-v+1,\cdots, m,\dots, m+v-1,m+v\right\}}}{J}_{i,x}^{\mathrm{\mathcal{E}},SP}, $$

where *n*_*c*_ is the number of consecutive significant SNPs in a window under AI, LOH/LCSH, or CNV/CNA, and ${J}_{i,x}^{\mathrm{\mathcal{E}},SP}=I\left[{\displaystyle {\sum}_{l=1, \dots,\ {n}_c}}{\displaystyle {\prod}_{z=x-{n}_c+l,x-{n}_c+l+1, \dots, x+l-1}}{I}_{i,z}^{\mathrm{\mathcal{E}},SP}>0\right]$, where the indicator function *I*[Δ_*i*_] equals unity if the event Δ_*i*_ is held; otherwise, it equals zero. If a SNP array also provides CN probes, the WAPs of the CN probes can be calculated similarly and independently of SNPs.

We proposed two procedures for evaluating the significance of a WAP statistic. The first procedure is a confidence interval method. First, we calculate the WAP statistics for AI, LOH/LCSH, and CNV/CNA for the test sample and all the normal reference samples. Next, we smoothen the WAPs using the local regression LOESS function [108] for every sample; ${\widetilde{W}}_{i,m}^{\mathcal{E},MP}\left(v,{n}_c\right)$ is used to indicate the smoothed WAP. If a SNP array also provides CN probes, the WAPs of the SNP and CN probes are arranged according to the order of their physical positions and then smoothed. Third, for each type of chromosomal aberration, the smoothed WAPs of all normal reference samples are ordered, and the *Q*%-quantile of the smoothed WAPs (denoted as ${\widetilde{Q}}_{i,m}^{\mathcal{E},MP}\left(v,{n}_c\right)$) are obtained (*Q*% = 95 %, 97.5 %, and 99 % in this paper). Finally, a region is detected as a chromosomal aberration if the smoothed WAP of the test sample exceeds the *Q*%-quantile of the smoothed WAP of all normal reference samples. Therefore, the first multipoint detector is defined as follows:

$$ {I}_{i,m}^{\mathcal{E},MP}\left(v,{n}_c,1\right)=I\left[{\widetilde {W}}_{i,m}^{\mathcal{E},MP}\left(v,{n}_c\right) > {\widetilde{Q}}_{i,m}^{\mathcal{E},MP}\left(v,{n}_c\right)\right]. $$

The second procedure is a hypothesis-testing method. First, we again calculate the WAP statistics for AI, LOH/LCSH, and CNV/CNA for the test sample and all the normal reference samples. Second, we calculate the mean and standard deviation of the WAP statistics for AI, LOH/LCSH, and CNV/CNA for all the normal reference samples (denoted as ${\hat{\mu}}_{i,m}^{\mathcal{E},MP}\left(v,{n}_c\right)$ and *S*_*i*,*m*_^ℰ,*MP*^(*v*, *n*_*c*_)). Finally, we conduct a hypothesis testing on the basis of the test statistic as follows:

$$ {Z}_{i,m}^{\mathrm{\mathcal{E}},MP}\left(v,{n}_c\right)=\frac{W_{i,m}^{\mathcal{E},MP}\left(v,{n}_c\right)-{\hat{\mu}}_{i,m}^{\mathrm{\mathcal{E}},MP}\left(v,{n}_c\right)+\frac{1}{M}\ }{S_{i,m}^{\mathrm{\mathcal{E}},MP}\left(v,{n}_c\right)}. $$

The adjusted *p* value after the Bonferroni correction is written as follows:

$$ {p}_{i,m}^{\mathrm{\mathcal{E}},MP}\left(v,{n}_c\right)= \min \left\{\left[1-\Phi \left({Z}_{i,m}^{\mathrm{\mathcal{E}},MP}\left(v,{n}_c\right)\right)\right]\cdot M,1\right\}. $$

Therefore, the second multipoint detector is defined as follows:

$$ {I}_{i,m}^{\mathrm{\mathcal{E}},MP}\left(v,{n}_c,2\right)=I\left[{p}_{i,m}^{\mathrm{\mathcal{E}},MP}\left(v,{n}_c\right) < 0.05\right] $$

In a real data analysis, users can consider *I*_*i*,*m*_^ℰ,*MP*^(*v*, *n*_*c*_, 1), *I*_*i*,*m*_^ℰ,*MP*^(*v*, *n*_*c*_, 2), or their combination *I*_*i*,*m*_^ℰ,*MP*^(*v*, *n*_***c***_) = *I*_*i*,*m*_^ℰ,*MP*^(*v*, *n*_*c*_, 1) × *I*_*i*,*m*_^ℰ,*MP*^(*v*, *n*_*c*_, 2).

### CN segmentation

ALICE provides two methods for CN segmentation: the original CBS algorithm [91, 92] and our proposed quick version of CBS. In the original CBS, all SNPs on a chromosome are arranged in the order of their physical positions. The starting and ending SNPs are connected to form a circle, where the connecting point is called 0. Two SNPs at physical positions A and B are chosen, where 0 < A < B. The two average HI values of SNPs in the region of A → B and the average HI value of SNPs in the region of B → 0 → A are calculated separately. A permutation test is used to analyze the difference in the two averages. If the difference is statistically significant, the region is partitioned into segments A → B, B → 0 and 0 → A. The procedure is recursively applied to all the segments until they cannot be further partitioned under some prespecified conditions.

Based on our experience, a large proportion of CNVs/CNAs are located in a region of AI and/or LOH/LCSH, and the CNV/CNA signal is more stable if a CNV/CNA is concomitant with AI and/or LOH/LCSH. We developed a quick CBS algorithm that uses the AI and LOH/LCSH signals from ALICE, which the original CBS algorithm did not use. The quick CBS performs segmentation procedures only in the regions of AI and LOH/LCSH. To give importance to the differential marker, the quick CBS assigns higher weights to SNPs that exhibit a stronger signal of chromosomal aberration. Let *d*_*i*,*m*_^ℰ^ = *W*_*i*,*m*_^ℰ,*MP*^(*v*, *n*_*c*_) − *Q*_*i*,*m*_^ℰ,*MP*^(*v*, *n*_*c*_) denote the difference in the WAP statistics between the test and normal reference samples. The difference is rescaled to a value between −1 and 1 as follows:

$$ {\widetilde{d}}_{i,m}^{\mathrm{\mathcal{E}}}=2\cdot \left({d}_{i,m}^{\mathrm{\mathcal{E}}} - {c}_{i, min}^{\mathrm{\mathcal{E}}}\right)/\left({c}_{i, max}^{\mathrm{\mathcal{E}}} - {c}_{i, min}^{\mathrm{\mathcal{E}}}\ \right)-1, $$

where *c*_*i*,*min*_^ℰ^ and *c*_*i*,*max*_^ℰ^ are the minimum and maximum of {*d*_*i*,*m*_^ℰ^, *m* = 1, ⋯, *M*}, respectively. The weight for the *m*^th^ SNP of the *i*^th^ individual is calculated as follows:

$$ {\widetilde{w}}_{i,m}=\frac{w_{i,m}}{{\displaystyle {\sum}_{m=1}^M}{w}_{i,m}}, $$

where $$ {w}_{i,m}={10}^{-10} + \max \left\{{\widetilde{d}}_{i,m}^{\mathcal{A}},{\widetilde{d}}_{i,m}^{\mathrm{\mathcal{L}}}\right\}\cdot I\left[ \max \left\{{\widetilde{d}}_{i,m}^{\mathcal{A}},{\widetilde{d}}_{i,m}^{\mathrm{\mathcal{L}}}\right\}>0\right]. $$

A weighted *t* test statistic based on weight ${\widetilde{w}}_{i,m}$ is used to analyze the difference in the averages of two segments in a region of AI or LOH/LCSH. A permutation test, which randomly shuffles the data in two segments, is used to calculate an empirical *p* value. If the empirical *p* value is significant, the region of AI or LOH/LCSH is segmented. It is noted that the quick CBS takes a long time if there are many small scattered regions of AI and LOH/LCSH. Hence, we recommend using the quick CBS when *b*/*a* < 5 %, where *a* denotes the total number of regions of AI and LOH/LCSH fragments on a chromosome and *b* denotes the number of regions of AI and LOH/LCSH fragments that are shorter than 1 % of the length of the same chromosome.

### Real-time qPCR

Primer pairs specific to genomic sequences of the candidate regions were designed using Primer Express (Applied Biosystems, Foster City, CA, USA), and the primer sequences are provided in Additional file 15. Quantitative genomic PCR was performed using SYBR green master mix (Applied Biosystems). For each reaction, 2 ng of gDNA was used as a template. The thermal cycling conditions were as follows: initial denaturation at 95 °C for 10 min, followed by 40 cycles of denaturation at 95 °C for 15 s, and combined annealing and extension at 60 °C for 1 min. The fluorescence signals were recorded at the end of the extension period of each cycle. The PCRs were performed on an ABI PRISM 7900 Sequence Detector (Applied Biosystems). All reactions were performed in triplicate. The *MPP4* gene locus on Chromosome 2 served as an internal control in this study. Let ${T}_{{\mathrm{target}}_i}$ and ${T}_{\mathrm{MPP}{4}_i}$ denote Ct values of the target gene and *MPP4* of the *i*^th^ replicate of the DNA sample in the test, where *i* = 1, 2, and 3 and *R*_target_ and *R*_MPP4_ denote the average Ct values of the target gene and *MPP4* of six healthy controls without any detectable CNVs/CNAs at the target regions, respectively. The estimated DNA CN and se of the target region in a haploid genome were obtained by calculating the mean and standard deviation of ${2}^{-\left[\left({T}_{{\mathrm{target}}_i}-{T}_{\mathrm{MPP}{4}_i}\right) - \left({R}_{\mathrm{target}}-{R}_{\mathrm{MPP}4}\right)\right]}$ over the triplicate samples.

## Availability of supporting data

The data sets supporting the results of this article are included within the article and its additional files. HI and genotype data of the samples in the International HapMap Project can be downloaded from the HapMap homepage (http://hapmap.ncbi.nlm.nih.gov/). ALICE and the used genomic reference databases can be downloaded from the ALICE homepage (http://hcyang.stat.sinica.edu.tw/software/ALICE.html).

## Additional files

Additional file 1: A whole-genome six-panel figure of the fifth sample in Fig. 3, which is genotyped using Axiom. This figure depicts the AF, AI, LOH/LCSH, and CNV/CNA analyses provided by ALICE. From top to bottom, the six-panel plot consists of the AF plot, AI plot, LOH/LCSH plot, HI and CN segmentation plot, proportion plot of CNV/CNA, and statistical significance plot of CNV/CNA. The details of each panel are described as follows: (1) In the AF plot, the vertical axis is the estimated AF, ranging from 0 to 1, and the horizontal axis is the physical position (Mb) on a chromosome. Each point denotes a SNP probe; blue and red points indicate non-AI and AI SNPs, respectively. (2) In the AI plot, the vertical axis is the proportion of AI SNPs, ranging from 0 to 1, and the horizontal axis is the physical position (Mb) on a chromosome. The light-red (deep-red) curve indicates the proportion of AI SNPs in sliding windows for the sample before (after) a smoothing spline. The light-blue (deep-blue) curve indicates the 95 % quantile of the proportions of AI SNPs in sliding windows for normal control samples before (after) a smoothing spline. The red bar at the top of the AI plot signifies a region of AI; thus, the deep-red curve is higher than the deep-blue curve. The deeper the red color in the bar, the higher is the proportion of AI SNPs in sliding windows. (3) In the LOH/LCSH plot, the vertical axis is the proportion of LOH/LCSH SNPs, ranging from 0 to 1, and the horizontal axis is the physical position (Mb) on a chromosome. The light-red (deep-red) curve indicates the proportion of LOH/LCSH SNPs in sliding windows for the sample before (after) a smoothing spline. The light-blue (deep-blue) curve indicates the 95 % quantile of the proportion of LOH/LCSH SNPs in sliding windows for normal control samples before (after) a smoothing spline. The red bar at the top of the LOH/LCSH plot signifies a region of LOH/LCSH; thus, the deep-red curve is higher than the deep-blue curve. The deeper the red color in the bar, the higher is the proportion of LOH/LCSH SNPs in sliding windows. (4) In the HI and CN segmentation plot, the vertical axis is the HI value, and the horizontal axis is the physical position (Mb) on a chromosome. Each point denotes a SNP probe (or a CN probe in Array 6.0); a light-blue point indicates the HI value of a marker, and a deep-blue point indicates the HI value of an AI SNP. Red and green segments obtained from the circular binary algorithm indicate segments of CN gain and loss, respectively. (5) In the proportion plot of CNV/CNA, the vertical axis is the proportion of CNV/CNA, ranging from 0 to 1, and the horizontal axis is the physical position (Mb) on a chromosome. Each point denotes a SNP probe (or a CN probe in Array 6.0). The light-red (deep-red) curve indicates the proportion of CN gain in sliding windows for the sample before (after) a smoothing spline. The light-green (deep-green) curve indicates the proportion of CN loss in sliding windows for the sample before (after) a smoothing spline. The light-blue (deep-blue) curve indicates the 95 % quantile of the proportions of CNV/CNA in sliding windows for normal control samples before (after) a smoothing spline. The green (red) bar at the top of this plot signifies a region that satisfies the following criteria: a) the proportion of CN loss (gain) in the sample is higher than that in the normal reference samples and b) *p* value of this region is statistically significant. The deeper the green (red) color in the bar, the higher is the proportion of CN loss (gain) in sliding windows. (6) In the statistical significance plot of CNV/CNA, the vertical axis is the adjusted *p* value (in a scale of − log_10_), and the horizontal axis is the physical position (Mb) on a chromosome. Each point denotes a SNP probe (or a CN probe in Array 6.0); a gray point represents the *p* value of a probe from a single-point association analysis. A blue point represents the *p* value from a multipoint association analysis, and the *p* value does not reach statistical significance. A green (red) point indicates a *p* value from a multipoint association analysis, and the *p* value reaches statistical significance of CN loss (gain). The green (red) bar at the top of this plot signifies a region that satisfies the following criteria: a) the proportion of CN loss (gain) in the sample is higher than that in the normal reference samples and (b) *p* value of this region is statistically significant. The deeper the green (red) color in the bar, the higher is the *p* value (in a scale of − log_10_) of CN loss (gain) in sliding windows. (TIFF 857 kb) Additional file 2: A whole-genome six-panel figure for the unpaired-sample analysis of a cancer cell line genotyped using Array 6.0. This figure shows the results in the unpaired-sample analysis of a pure cancer cell line sample genotyped using Array 6.0. From top to bottom, the six-panel plot consists of the AF plot, AI plot, LOH/LCSH plot, HI and CN segmentation plot, proportion plot of CNV/CNA, and statistical significance plot of CNV/CNA. The details of the illustrations of each plot are provided in Additional file 1. (TIFF 1223 kb) Additional file 3: Relationship between the length of the overlapped region and the overlap ratio. The vertical axis is the overlap ratio (%), and the horizontal axis is the length of the overlapped region (Mb). Each point denotes a region that was identified by Axiom and overlapped with the region identified by Array 6.0. The red line is a regression curve estimated by a local polynomial regression fitting. The histogram in the bottom and histogram in the right-hand side summarize frequency distributions of the length of the overlapped region and the overlap ratio, respectively. (TIFF 204 kb) Additional file 4: Dynamic patterns of HI of the admixed samples obtained using an unpaired-sample multipoint analysis. This figure provides a whole-genome HI plot of the admixed samples genotyped using Axiom and analyzed using unpaired multipoint detection. From top to bottom, each panel represents an admixed sample with a proportion of 0 %–100 % of cancer cell line DNA, with an increment of 10 %. In each HI plot, the vertical axis is the value of HI, and the horizontal axis is the physical position (Mb) on a chromosome. A red point indicates a SNP probe that detected a CN gain with AI and/or LOH; a green point indicates a SNP probe that detected a CN loss with AI and/or LOH; and a yellow point indicates a SNP probe without CNV/CNA but with AI and/or LOH. The SNPs not included in the abovementioned situations are colored in gray. (TIFF 5891 kb) Additional file 5: Dynamic patterns of AF of the admixed samples analyzed using an unpaired-sample multipoint analysis. This figure provides a whole-genome AF plot of the admixed samples genotyped using Axiom and analyzed using unpaired multi-point detection. From top to bottom, each panel represents an admixed sample with a proportion of 0 %–100 % of cancer cell line DNA, with an increment of 10 %. In each AF plot, the vertical axis is the value of AF, and the horizontal axis is the physical position (Mb) on a chromosome. A red point indicates a SNP probe that detected a CN gain with AI and/or LOH; a green point indicates a SNP probe that detected a CN loss with AI and/or LOH; and a yellow point indicates a SNP probe without CNV/CNA but with AI and/or LOH. The SNPs not included in the abovementioned situations are colored in gray. (TIFF 8220 kb) Additional file 6: A six-panel figure for the paired-sample analysis of a cancer cell line genotyped using Axiom array. This figure shows the results in the paired-sample analysis of a pure cancer cell line sample compared with that of the corresponding normal blood cell line. From top to bottom, the six-panel plot consists of the AF plot, AI plot, LOH/LCSH plot, HI and CN segmentation plot, proportion plot of CNV/CNA, and statistical significance plot of CNV/CNA. The details of the illustrations of each plot are provided in Additional file 1. (TIFF 1144 kb) Additional file 7: Dynamic patterns of HI of the admixed samples analyzed using a paired-sample multipoint analysis. This figure provides a whole-genome HI plot of the admixed samples genotyped using Axiom and analyzed using paired multipoint detection. From top to bottom, each panel represents an admixed sample with a proportion of 0 %–100 % of cancer cell line DNA, with an increment of 10 %. In each HI plot, the vertical axis is the value of HI, and the horizontal axis is the physical position (Mb) on a chromosome. A red point indicates a SNP probe that detected a CN gain with AI and/or LOH; a green point indicates a SNP probe that detected a CN loss with AI and/or LOH; and a yellow point indicates a SNP probe without CNV/CNA but with AI and/or LOH. The SNPs not included in the abovementioned situations are colored in gray. (TIFF 5737 kb) Additional file 8: Dynamic patterns of AF of the admixed samples analyzed using a paired-sample multipoint analysis. This figure provides a whole-genome AF plot of the admixed samples genotyped using Axiom and analyzed using paired multipoint detection. From top to bottom, each panel represents an admixed sample with a proportion of 0 %–100 % of cancer cell line DNA, with an increment of 10 %. In each AF plot, the vertical axis is the value of AF, and the horizontal axis is the physical position (Mb) on a chromosome. A red point indicates a SNP probe that detected a CN gain with AI and/or LOH; a green point indicates a SNP probe that detected a CN loss with AI and/or LOH; and a yellow point indicates a SNP probe without CNV/CNA but with AI and/or LOH. The SNPs not included in the abovementioned situations are colored in gray. (TIFF 8015 kb) Additional file 9: Paired-sample analysis. (DOCX 270 kb) Additional file 10: An example of a similarity in segmentation detection using the original and quick CBS algorithms. This figure depicts the AF plot, LOH/LCSH plot, and two HI and CN segmentation plots obtained using the original and quick CBS algorithms. (a) The results of Chromosome 2 of the 11^th^ sample in Fig. 3. An LCSH region, from 0.66 to 22.57 Mb, which was identified using ALICE, is marked by a yellow rectangle. (b) The results of the LCSH region from 0.66 to 22.57 Mb of Chromosome 2 of the 11^th^ sample in Fig. 3. (TIFF 1975 kb) Additional file 11: The interface of ALICE software—The component “Main Functions”. (TIFF 212 kb) Additional file 12: The interface of ALICE software—The component “Genome Browser”. (TIFF 167 kb) Additional file 13: The interface of ALICE software—The component “Aberration Integration”. (TIFF 178 kb) Additional file 14: The cross-sample plot of the multipoint LOH/LCSH analyses of the three samples used in Fig. 5. The plot comprises four panels: (a) The top-left panel is a cross-sample and cross-chromosome plot. The vertical axis is the index of study samples, and the horizontal axis is the physical position (Mb) on each of the 23 chromosomes. The blue and red bars represent SNPs without and with LOH/LSCH, respectively. (b) The top-right panel is a histogram of cross-chromosome aberration frequency. The vertical axis is the index of study samples, and the horizontal axis is the cross-chromosome aberration frequency of the corresponding samples. The pink (skyblue) background represents that the genetic gender of a sample is female (male). The histogram represents the aberration frequency of LOH/LCSH SNPs across the chromosomes of the corresponding samples. (c) The bottom-left panel is a histogram of the cross-sample aberration frequency. The vertical axis is the cross-sample aberration frequency of a SNP, and the horizontal axis is the physical position (Mb) on each of the 23 chromosomes. The purple line represents the aberration proportion of samples carrying the SNPs with LOH/LCSH. (d) The bottom-right panel is the legend of the genetic gender that is used in panel (b), where the pink (skyblue) background represents that the genetic gender of a sample is female (male). (TIFF 1656 kb) Additional file 15: Sequence information of the primers used for qPCR. (XLSX 11 kb)

## Abbreviations

a-CGH: Array-comparative genomic hybridization
AF: Allele frequency
AI: Allelic imbalance
ALICE: AF/LOH/LCSH/AI/CNV/CNA Enterprise
CBS: Circular binary segmentation
CN: Copy number
CNA: Copy number alteration
CNV: Copy number variation
CPA: Coefficient of preferential amplification or hybridization
FPR: False positive rate
HI: Hybridization intensity
LCSH: Long contiguous stretches of homozygosity
LIM: Linear interpolation method
LOH: Loss of heterozygosity
SNP: Single nucleotide polymorphism
SNV: Single nucleotide variation
TPR: True positive rate
