# Supplementary figures and images for: An integrated analysis tool for analyzing hybridization intensities and genotypes using new-generation population-optimized human arrays
Source: BMC Genomics. 2016 Mar 31;17:266. doi: 10.1186/s12864-016-2478-8 (PMC4815280; doi:10.1186/s12864-016-2478-8)

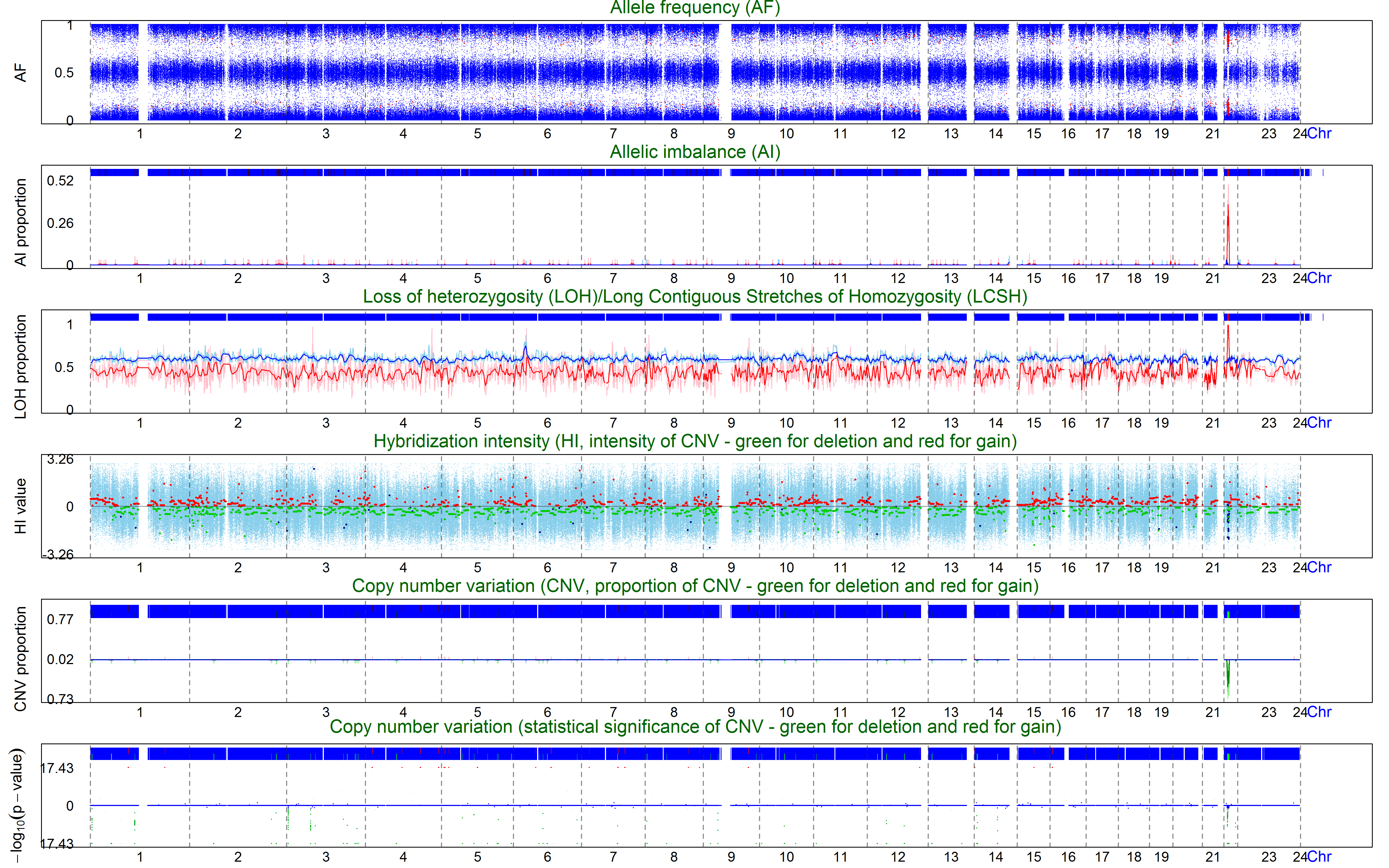

Supplement: Additional file 1: — A whole-genome six-panel figure of the fifth sample in Fig. 3, which is genotyped using Axiom. This figure depicts the AF, AI, LOH/LCSH, and CNV/CNA analyses provided by ALICE. From top to bottom, the six-panel plot consists of the AF plot, AI plot, LOH/LCSH plot, HI and CN segmentation plot, proportion plot of CNV/CNA, and statistical significance plot of CNV/CNA. The details of each panel are described as follows: (1) In the AF plot, the vertical axis is the estimated AF, ranging from 0 to 1, and the horizontal axis is the physical position (Mb) on a chromosome. Each point denotes a SNP probe; blue and red points indicate non-AI and AI SNPs, respectively. (2) In the AI plot, the vertical axis is the proportion of AI SNPs, ranging from 0 to 1, and the horizontal axis is the physical position (Mb) on a chromosome. The light-red (deep-red) curve indicates the proportion of AI SNPs in sliding windows for the sample before (after) a smoothing spline. The light-blue (deep-blue) curve indicates the 95 % quantile of the proportions of AI SNPs in sliding windows for normal control samples before (after) a smoothing spline. The red bar at the top of the AI plot signifies a region of AI; thus, the deep-red curve is higher than the deep-blue curve. The deeper the red color in the bar, the higher is the proportion of AI SNPs in sliding windows. (3) In the LOH/LCSH plot, the vertical axis is the proportion of LOH/LCSH SNPs, ranging from 0 to 1, and the horizontal axis is the physical position (Mb) on a chromosome. The light-red (deep-red) curve indicates the proportion of LOH/LCSH SNPs in sliding windows for the sample before (after) a smoothing spline. The light-blue (deep-blue) curve indicates the 95 % quantile of the proportion of LOH/LCSH SNPs in sliding windows for normal control samples before (after) a smoothing spline. The red bar at the top of the LOH/LCSH plot signifies a region of LOH/LCSH; thus, the deep-red curve is higher than the deep-blue curve. The deeper the r [file 12864_2016_2478_MOESM1_ESM.tiff]

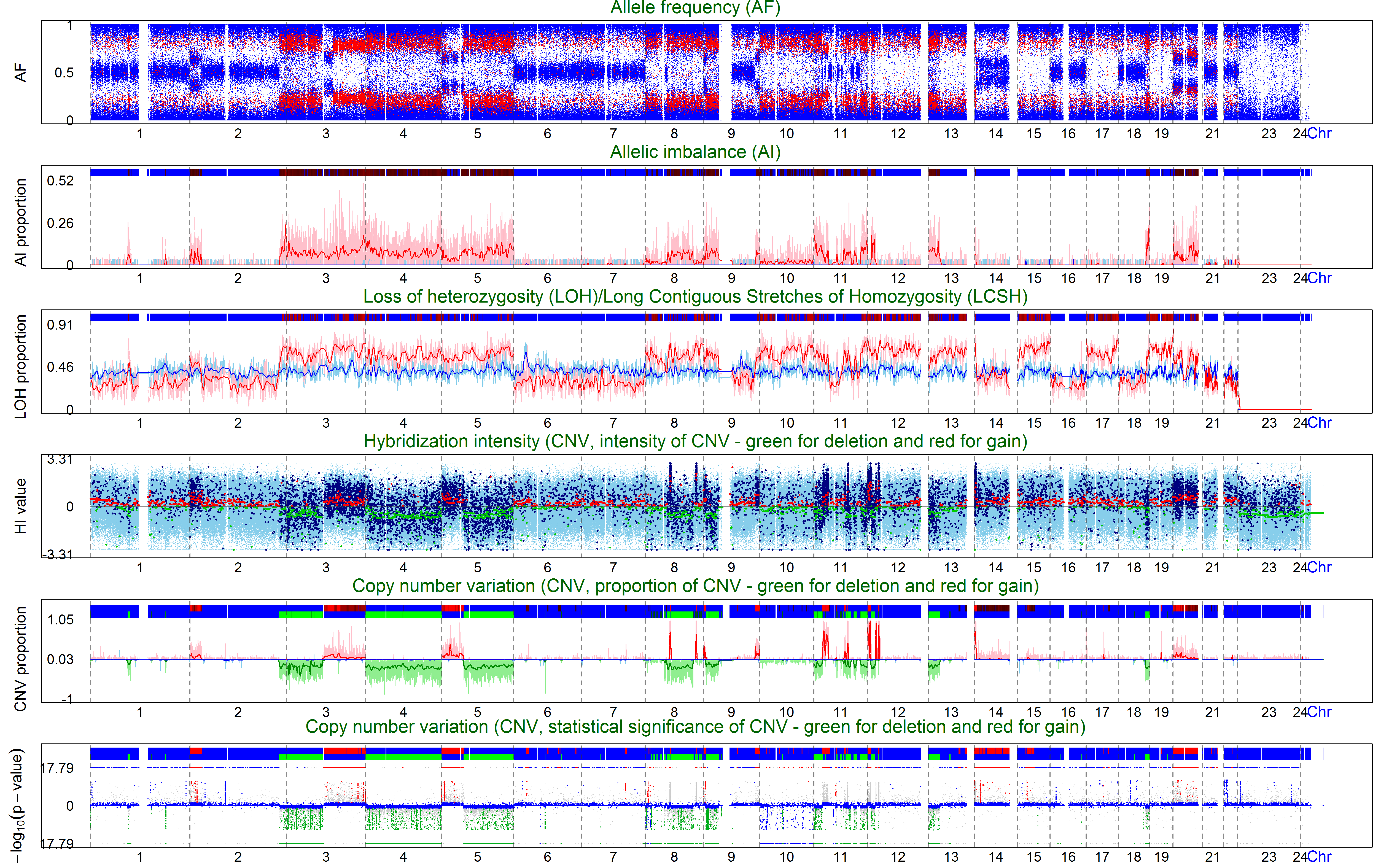

Supplement: Additional file 2: — A whole-genome six-panel figure for the unpaired-sample analysis of a cancer cell line genotyped using Array 6.0. This figure shows the results in the unpaired-sample analysis of a pure cancer cell line sample genotyped using Array 6.0. From top to bottom, the six-panel plot consists of the AF plot, AI plot, LOH/LCSH plot, HI and CN segmentation plot, proportion plot of CNV/CNA, and statistical significance plot of CNV/CNA. The details of the illustrations of each plot are provided in Additional file 1. (TIFF 1223 kb) [file 12864_2016_2478_MOESM2_ESM.tiff]

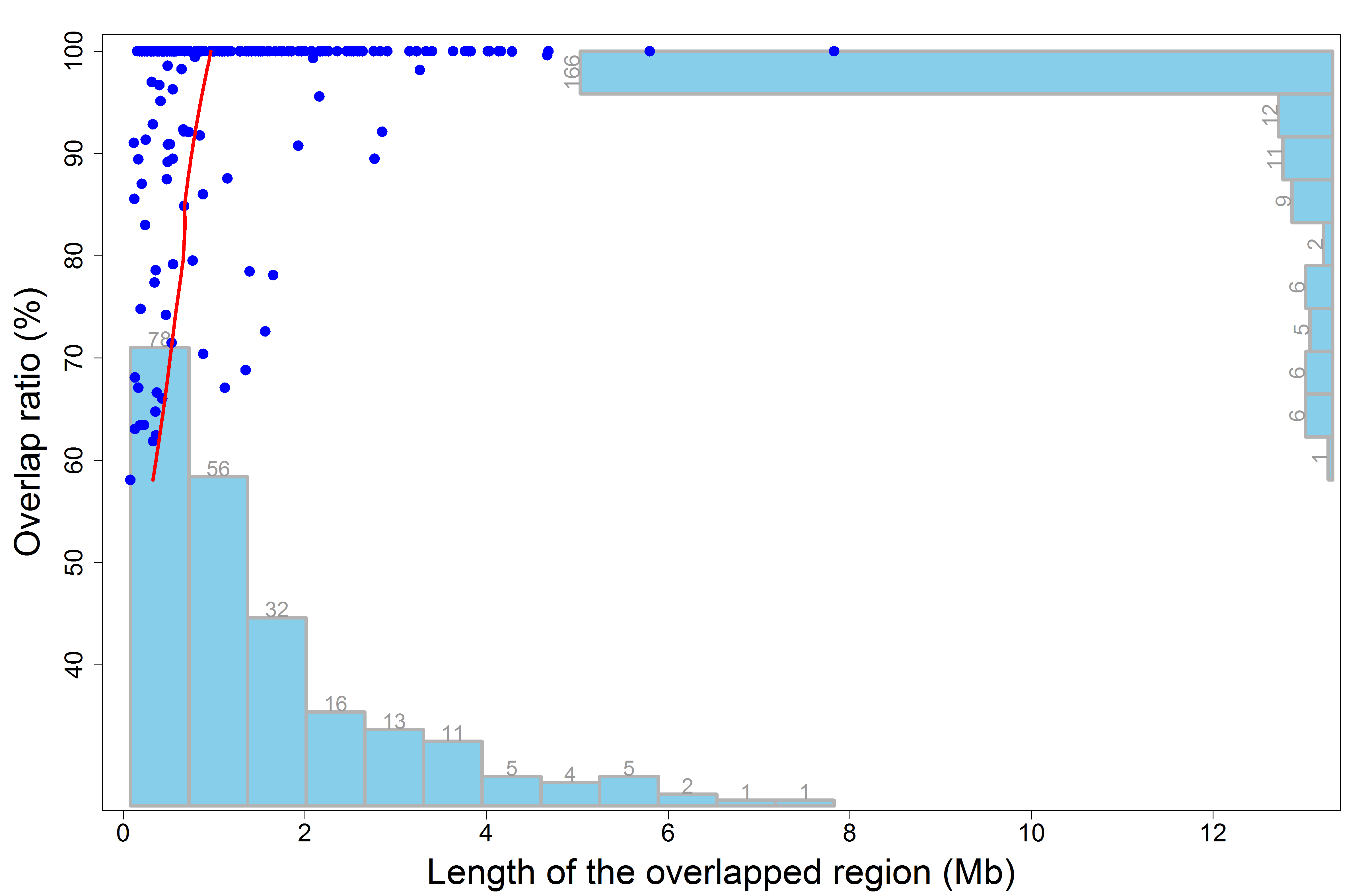

Supplement: Additional file 3: — Relationship between the length of the overlapped region and the overlap ratio. The vertical axis is the overlap ratio (%), and the horizontal axis is the length of the overlapped region (Mb). Each point denotes a region that was identified by Axiom and overlapped with the region identified by Array 6.0. The red line is a regression curve estimated by a local polynomial regression fitting. The histogram in the bottom and histogram in the right-hand side summarize frequency distributions of the length of the overlapped region and the overlap ratio, respectively. (TIFF 204 kb) [file 12864_2016_2478_MOESM3_ESM.tiff]

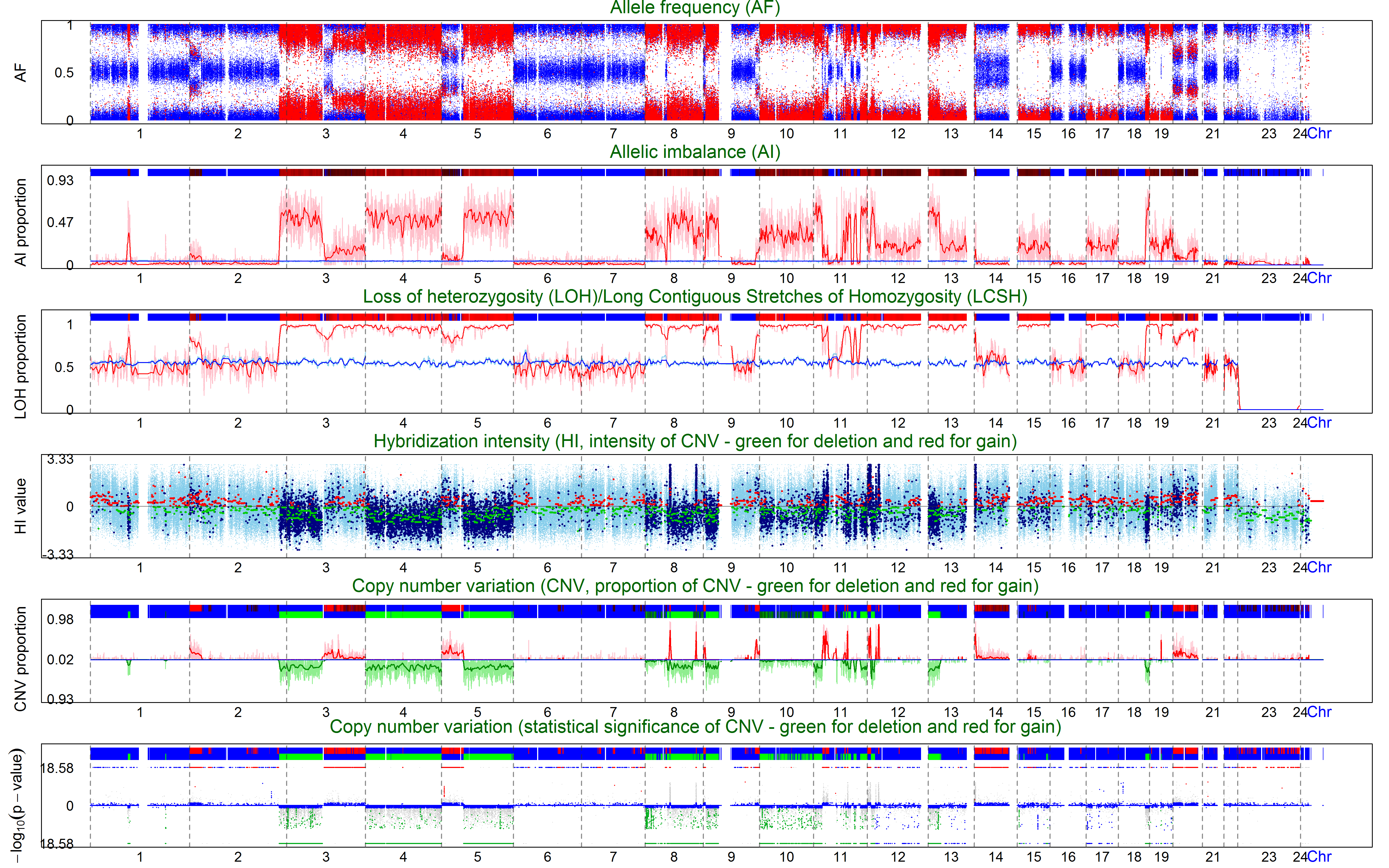

Supplement: Additional file 6: — A six-panel figure for the paired-sample analysis of a cancer cell line genotyped using Axiom array. This figure shows the results in the paired-sample analysis of a pure cancer cell line sample compared with that of the corresponding normal blood cell line. From top to bottom, the six-panel plot consists of the AF plot, AI plot, LOH/LCSH plot, HI and CN segmentation plot, proportion plot of CNV/CNA, and statistical significance plot of CNV/CNA. The details of the illustrations of each plot are provided in Additional file 1. (TIFF 1144 kb) [file 12864_2016_2478_MOESM6_ESM.tiff]

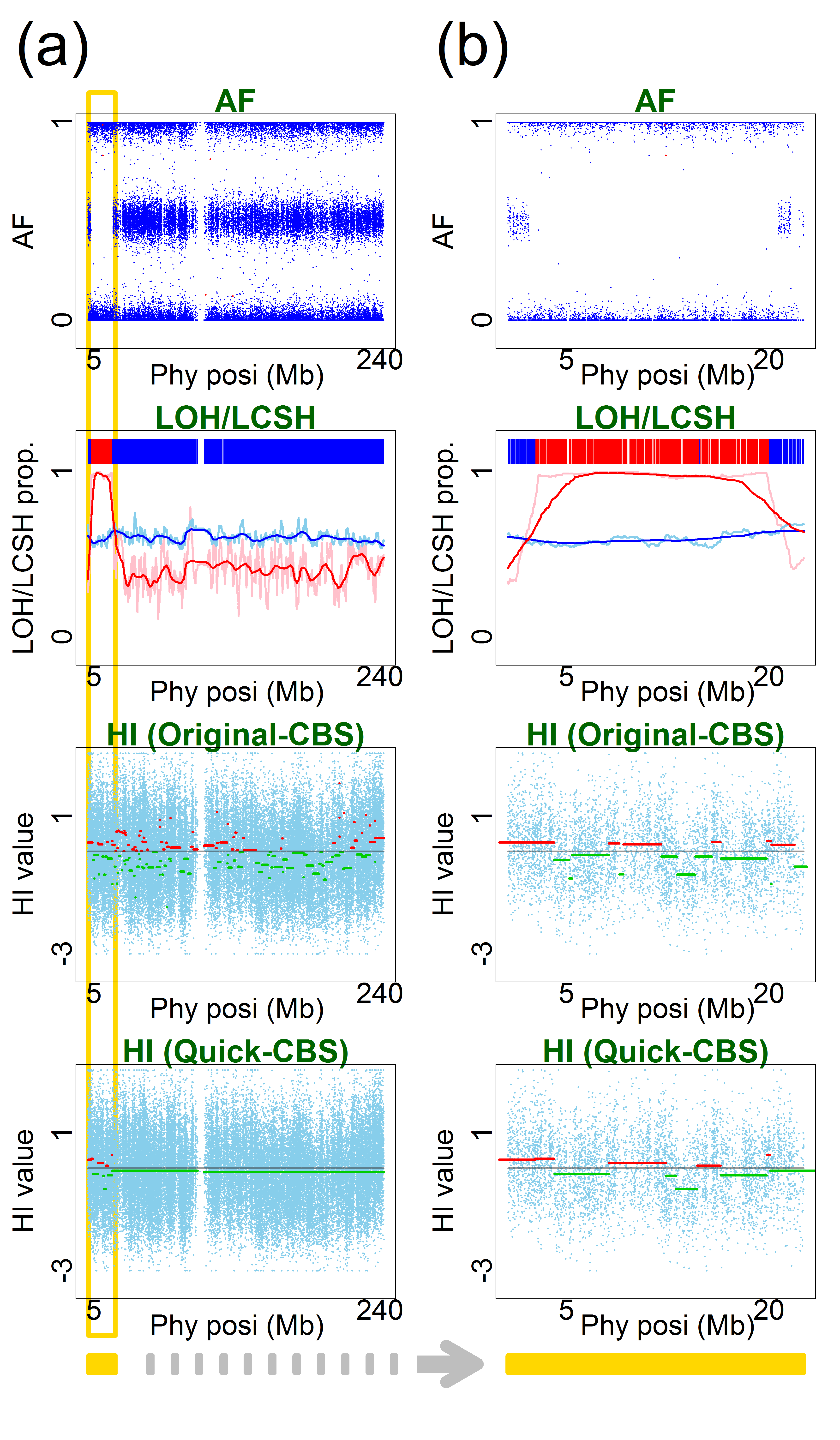

Supplement: Additional file 10: — An example of a similarity in segmentation detection using the original and quick CBS algorithms. This figure depicts the AF plot, LOH/LCSH plot, and two HI and CN segmentation plots obtained using the original and quick CBS algorithms. (a) The results of Chromosome 2 of the 11th sample in Fig. 3. An LCSH region, from 0.66 to 22.57 Mb, which was identified using ALICE, is marked by a yellow rectangle. (b) The results of the LCSH region from 0.66 to 22.57 Mb of Chromosome 2 of the 11th sample in Fig. 3. (TIFF 1975 kb) [file 12864_2016_2478_MOESM10_ESM.tiff]

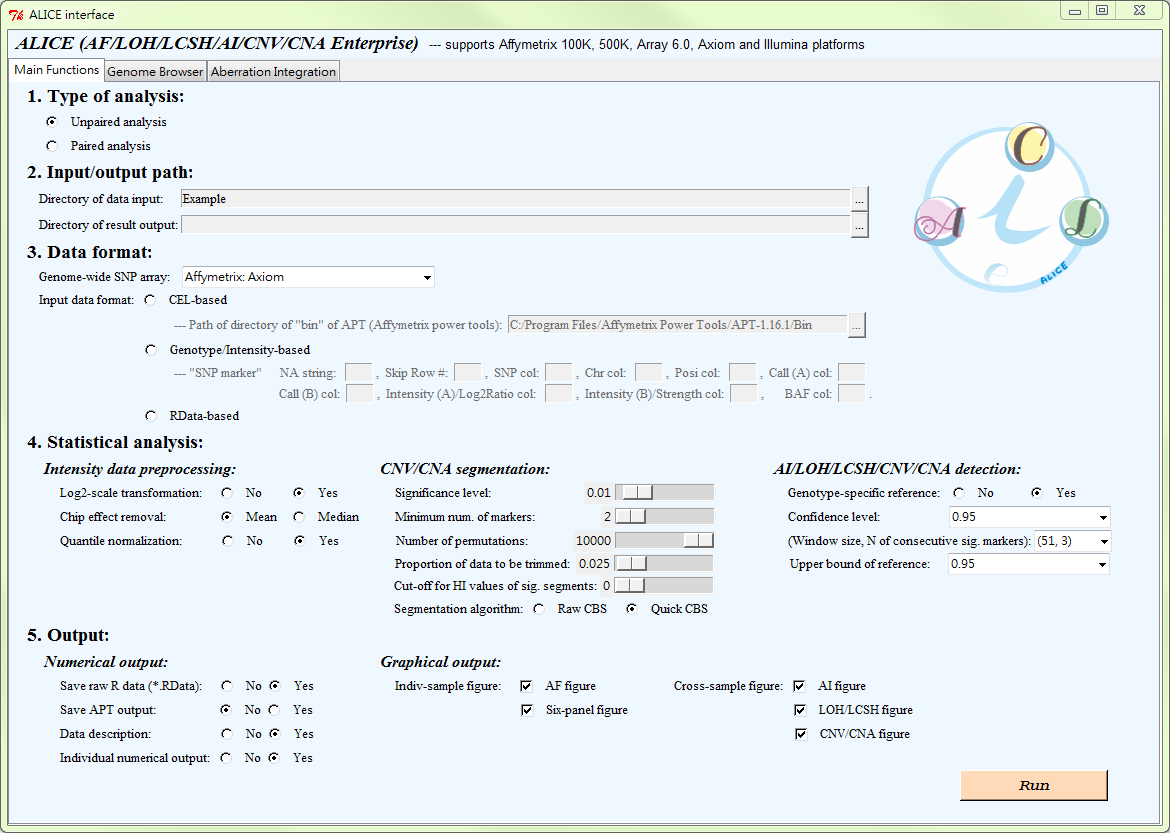

Supplement: Additional file 11: — The interface of ALICE software—The component “Main Functions”. (TIFF 212 kb) [file 12864_2016_2478_MOESM11_ESM.tiff]

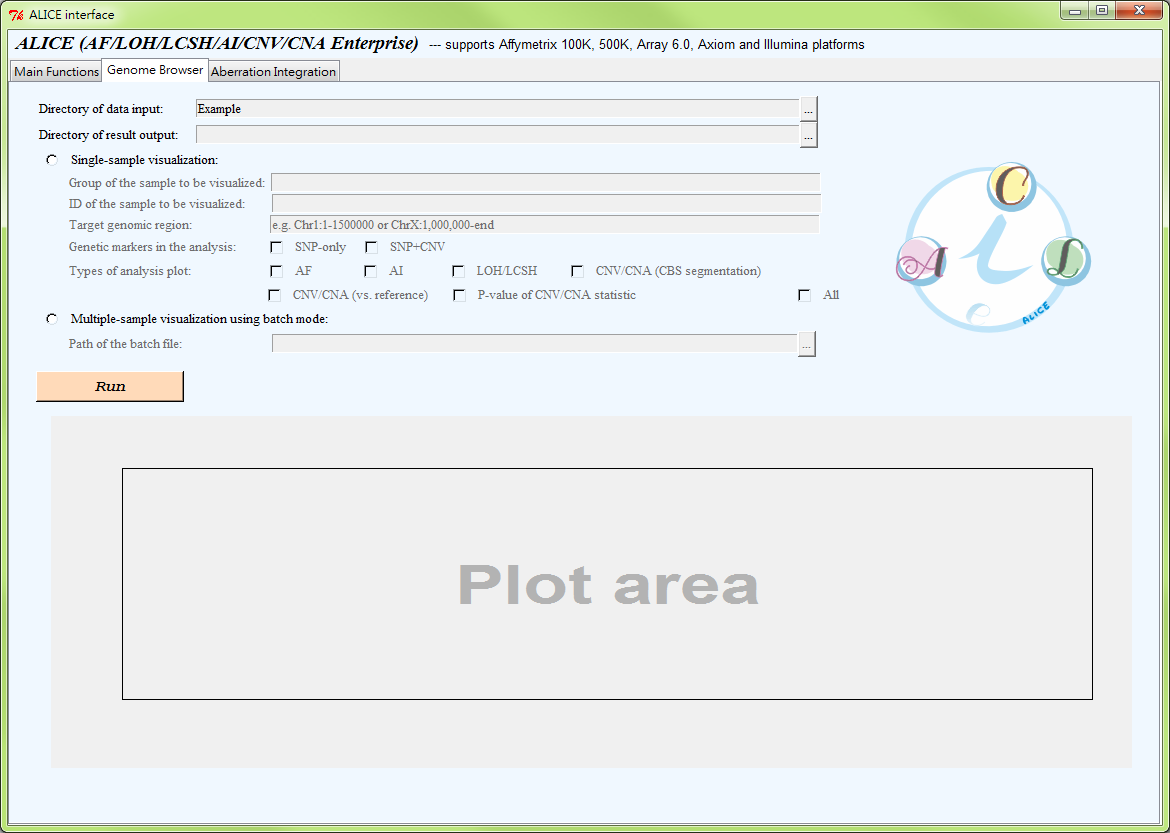

Supplement: Additional file 12: — The interface of ALICE software—The component “Genome Browser”. (TIFF 167 kb) [file 12864_2016_2478_MOESM12_ESM.tiff]

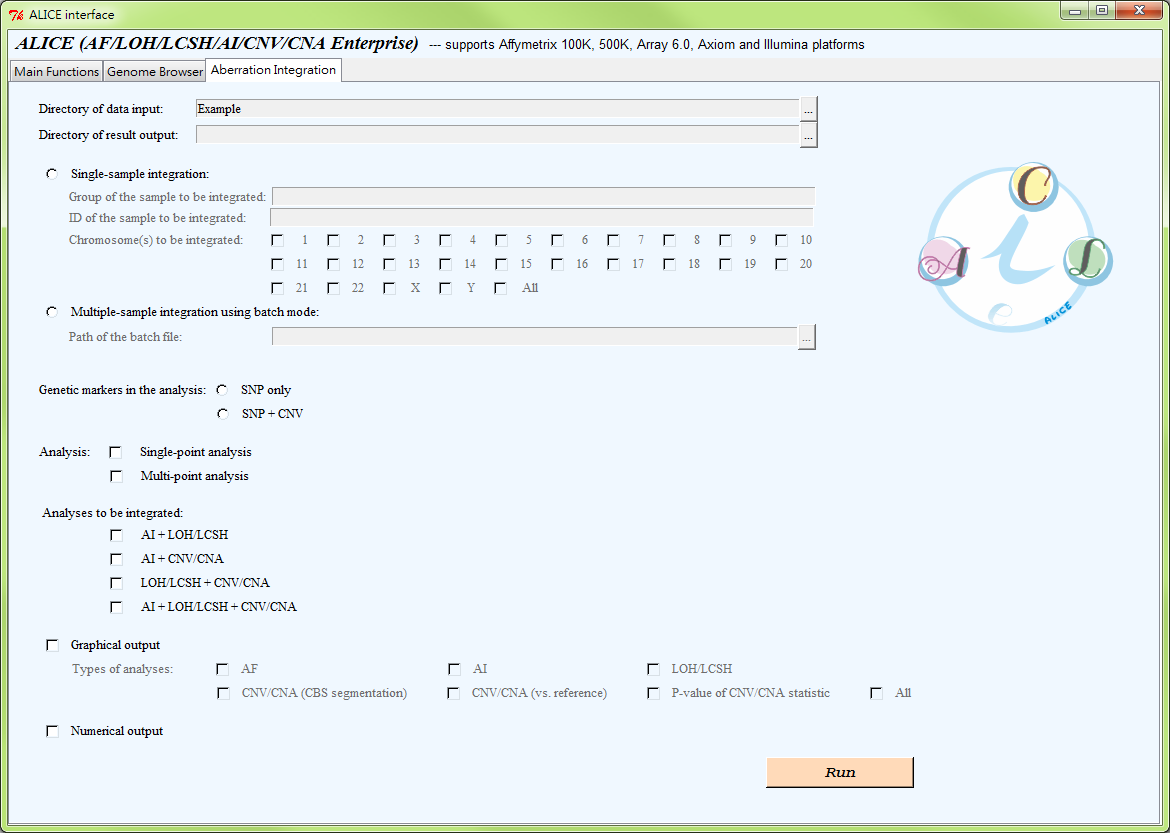

Supplement: Additional file 13: — The interface of ALICE software—The component “Aberration Integration”. (TIFF 178 kb) [file 12864_2016_2478_MOESM13_ESM.tiff]

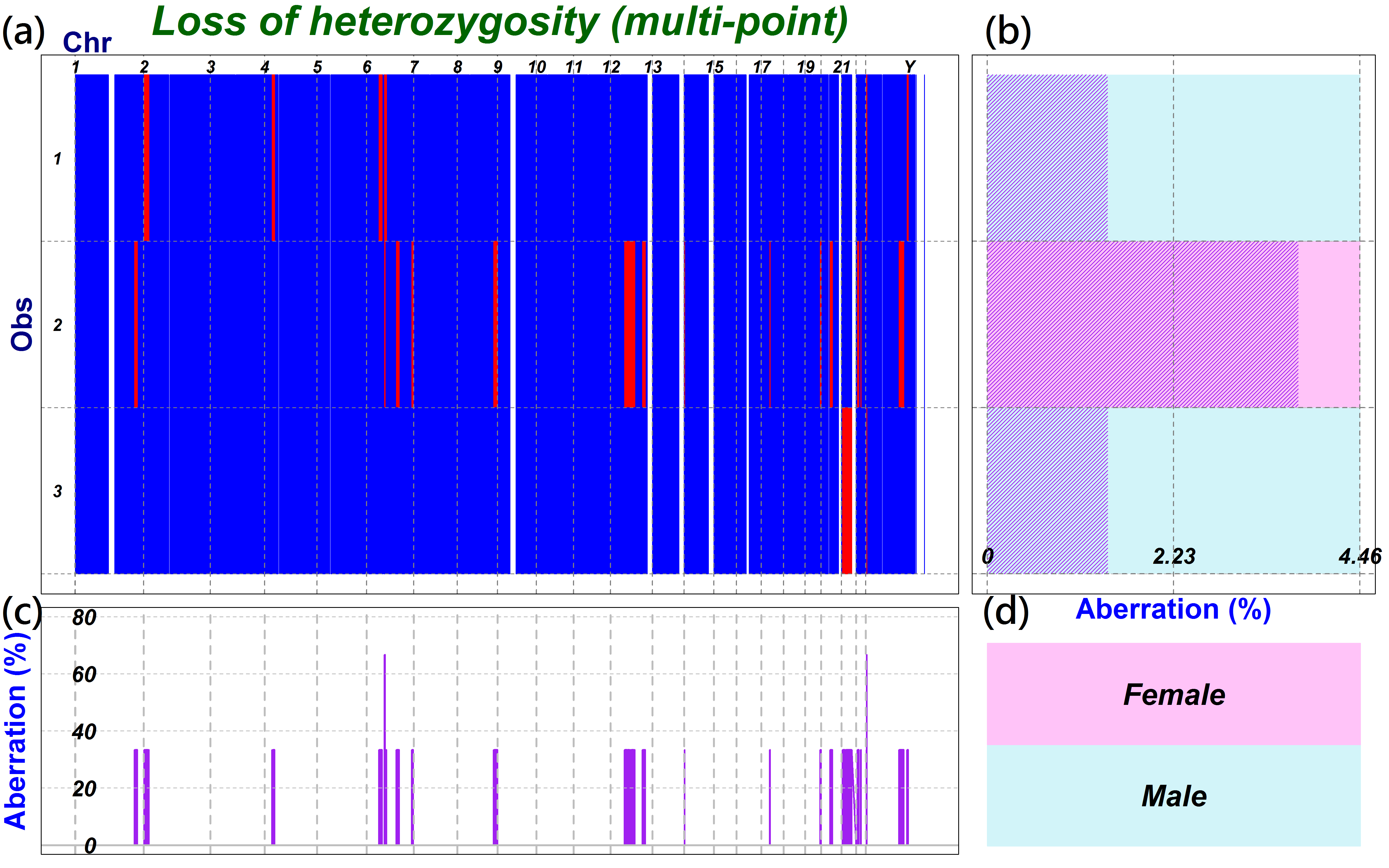

Supplement: Additional file 14: — The cross-sample plot of the multipoint LOH/LCSH analyses of the three samples used in Fig. 5. The plot comprises four panels: (a) The top-left panel is a cross-sample and cross-chromosome plot. The vertical axis is the index of study samples, and the horizontal axis is the physical position (Mb) on each of the 23 chromosomes. The blue and red bars represent SNPs without and with LOH/LSCH, respectively. (b) The top-right panel is a histogram of cross-chromosome aberration frequency. The vertical axis is the index of study samples, and the horizontal axis is the cross-chromosome aberration frequency of the corresponding samples. The pink (skyblue) background represents that the genetic gender of a sample is female (male). The histogram represents the aberration frequency of LOH/LCSH SNPs across the chromosomes of the corresponding samples. (c) The bottom-left panel is a histogram of the cross-sample aberration frequency. The vertical axis is the cross-sample aberration frequency of a SNP, and the horizontal axis is the physical position (Mb) on each of the 23 chromosomes. The purple line represents the aberration proportion of samples carrying the SNPs with LOH/LCSH. (d) The bottom-right panel is the legend of the genetic gender that is used in panel (b), where the pink (skyblue) background represents that the genetic gender of a sample is female (male). (TIFF 1656 kb) [file 12864_2016_2478_MOESM14_ESM.tiff]
