# Supplementary material for: An integrated analysis tool for analyzing hybridization intensities and genotypes using new-generation population-optimized human arrays
Source: BMC Genomics. 2016 Mar 31;17:266. doi: 10.1186/s12864-016-2478-8 (PMC4815280; doi:10.1186/s12864-016-2478-8)
Supplement: Additional file 9: — Paired-sample analysis. (DOCX 270 kb) [file 12864_2016_2478_MOESM9_ESM.docx]

**Additional file 9.** **Paired-sample analysis****.**

Similar to the unpaired-sample analysis, we deciphered the interrelationship between **the successful detection of chromosomal aberrations** $S(p)$ and **three influential factors: (1) admixture proportion, (2) mean** difference in HI values between the study sample and normal reference**, and (3) the length of the region of CNVs/CNAs. We** also investigated how $p_{min}$ **relates to** the difference in HI values **and length of the region of CNVs/CNAs.**

First, similar to the unpaired-sample analysis, the successful detection rate $S(p)$ in this paired-sample analysis also increased with an increase in the admixture proportion $p$% in general. For a CN gain, $S(p)$ increased from 6.26 to 91.4% as $p$% was increased from 10 to 90% (pink right hatched bars in Fig. A). **The increasing trend of** $S(p)$ had a positive slope coefficient of 1.55 × 10^−2^ (se = 5.30 × 10^−3^), and the *p* value was 3.69 × 10^−3^ for the linear regression of $S(p)$ on $p$%. For a CN loss, $S(p)$ increased from 1.29 to 94.61% as $p$% was increased from 10 to 90% (green left hatched bars in Fig. A). **The increasing trend of** $S(p)$ had a slope coefficient of 3.37 × 10^−2^ (se = 4.82 × 10^−3^), and the *p* value was 9.51 × 10^−12^ for the linear regression.

Second, $S(p)$ was positively and negatively correlated with the differences in HI values for CN gain and loss, respectively. For a CN gain, $S(p)$ increased with an increase in the difference in HI values. **The increasing trend of** $S(p)$ had a positive slope coefficient of 3.29 (se = 4.86 × 10^−1^), and the *p* value was 4.20 × 10^−11^ for the linear regression of $S(p)$ on the difference in HI values. For a CN loss, $S(p)$ increased as the difference in HI values became more negative, with a positive slope coefficient of 14.64 (se = 7.79 × 10^−1^) and a *p* value of 7.74 × 10^−60^ for the linear regression.

Finally, $S(p)$ was positively correlated with **the length of the region of CNVs/CNAs**. For a CN gain, $S(p)$ increased with an increase in the length of the region of **CNVs/CNAs**; **the increasing trend of** $S(p)$ had a positive slope coefficient of 0.35 (se = 2.99 × 10^−2^), and the *p* value was 1.84 × 10^−27^. For a CN loss as well, $S(p)$ increased with an increase in the length of the region of **CNVs/CNAs**; **the increasing trend of** $S(p)$ had a positive slope coefficient of 0.11 (se = 1.07 × 10^−2^), and the *p* value was 1.69 × 10^−22^.

Similar to the unpaired-sample analysis, **the minimum admixture proportion** ($p_{min}$%) was related to the difference in HI values **and length of the region of CNVs/CNAs. The results first showed that** $p_{min}$ was **negatively** correlated with the difference in HI values. For a CN gain, $p_{min}$ decreased with an increase in the difference in HI values (Spearman correlation coefficient was −0.60, with a *p* value of 1.03 × 10^−13^). For a CN loss, $p_{min}$ decreased as the difference in HI values became more negative (Spearman correlation coefficient was −0.70, with a *p* value of 1.78 × 10^−42^). Second, $p_{min}$ was negatively correlated with the length of the region of **CNVs/CNAs** when the admixture proportion $p$% was higher than 50% (Fig. A). For a CN gain, $p_{min}$ decreased with an increase in the length of the region of **CNVs/CNAs** (Spearman correlation coefficient was −0.39, with a *p* value of 6.41 × 10^−6^). Similarly, for a CN loss, $p_{min}$ decreased with an increase in the length of the region of **CNVs/CNAs** (Spearman correlation coefficient was −0.63, with a *p* value of 3.66 × 10^−32^).


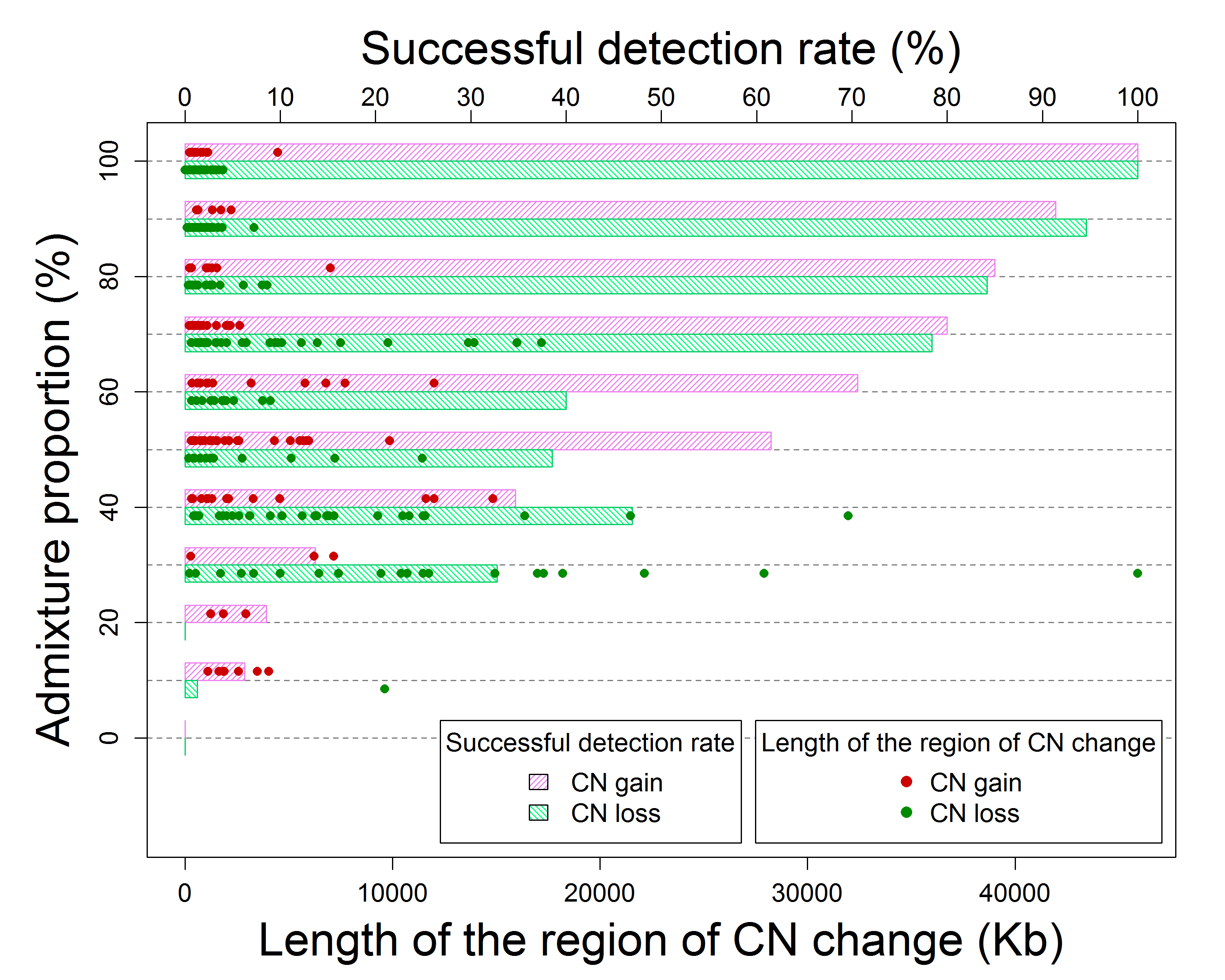


**Fig. A. Successful detection rates of CN detection in the admixed samples analyzed using a paired-sample analysis. The vertical axis indicates the admixture proportion (%), and the horizontal axis is the successful detection rate (%) (top) and the length of the region of CN loss and gain (Kb) (bottom) in the pure cancer cell line sample. Under an admixture proportion, the proportion of successfully detected regions among all 276 (127) regions of CN gain (loss) is presented by a red (green) hatched bar, where the value on the bar represents the coordinate of the top horizontal axis. For each of the 276 (127) regions of CN gain (loss), the minimum admixture proportions that were successfully detected are presented by red (green) points, where the value of the minimum admixture proportion is represented by the coordinate of the vertical axis, and length (Kb) of the region by the coordinate of the bottom horizontal axis.**
